# Chronic Corticosterone Exposure Suppresses Copper Transport through GR-Mediated Intestinal CTR1 Pathway in Mice

**DOI:** 10.3390/biology12020197

**Published:** 2023-01-28

**Authors:** Shihui Guo, Zijin Chen, Yingying Dong, Yingdong Ni, Ruqian Zhao, Wenqiang Ma

**Affiliations:** 1 Key Laboratory of Animal Physiology and Biochemistry, Ministry of Agriculture and Rural Affairs, College of Veterinary Medicine, Nanjing Agricultural University, Nanjing 210095, China; 2 MOE Joint International Research Laboratory of Animal Health & Food Safety, Nanjing Agricultural University, Nanjing 210095, China

**Keywords:** corticosterone, copper, glucocorticoid receptor, mice

## Abstract

**Simple Summary:**

Copper (Cu) is a redox active metal and an essential trace element for human health, which is required as a cofactor for enzymes involved in numerous important cellular functions and pathways. Chronic stress is a common environmental issue, which induces nutritional or metabolic disorders and leads to a significant adverse health consequence. In this study, corticosterone drinking water was used to simulate chronic stress to explore the effects of stress on the disorder of copper metabolism and its regulatory mechanism in mice. It was proved that corticosterone impairs copper transport by down-regulating intestinal CTR1 expression via GR-mediated transcriptional inhibition in vivo and in vitro. This study provides the theoretical basis for regulating the copper homeostasis under chronic stress.

**Abstract:**

Numerous studies have discovered that chronic stress induces metabolic disorders by affecting iron and zinc metabolism, but the relationship between chronic stress and copper metabolism remains unclear. Here, we explore the influence of chronic corticosterone (CORT) exposure on copper metabolism and its regulatory mechanism in mice. Mice were treated with 100 μg/mL CORT in drinking water for a 4-week trial. We found that CORT treatment resulted in a significant decrease in plasma copper level, plasma ceruloplasmin activity, plasma and liver Cu/Zn-SOD activity, hepatic copper content, and liver metallothionein content in mice. CORT treatment led to the reduction in duodenal expression of copper transporter 1 (CTR1), duodenal cytochrome b (DCYTB), and ATPase copper-transporting alpha (ATP7A) at the mRNA and protein level in mice. CORT treatment activated nuclear glucocorticoid receptor (GR) and down-regulated CRT1 expression in Caco-2 cells, whereas these phenotypes were reversible by an antagonist of GR, RU486. Chromatin immunoprecipitation analysis revealed that GR bound to the *Ctr1* promoter in Caco-2 cells. Transient transfection assays in Caco-2 cells demonstrated that the *Ctr1* promoter was responsive to the CORT-activated glucocorticoid receptor, whereas mutation/deletion of the glucocorticoid receptor element (GRE) markedly impaired activation of the *Ctr1* promoter. In addition, CORT-induced downregulation of *Ctr1* promoter activity was markedly attenuated in Caco-2 cells when RU486 was added. These findings present a novel molecular target for CORT that down-regulates intestinal CTR1 expression via GR-mediated trans-repression in mice.

## 1. Introduction

Copper (Cu) is one of the essential trace elements and plays a key role in respiration, antioxidant activity, tissue integrity, and synaptic function [1,2]. Well-balanced copper homeostasis is essential for health, whereas disruption of copper homeostasis results in pathological outcomes. Numerous studies have reported that copper deficiency disrupts iron metabolism, produces anemia-like symptoms, impairs growth performance, increases incidence of infections, hyperlipidemia and coronary heart disease, and decreases glucose tolerance [3,4]. Moreover, copper overload is associated with tissue damage due to redox cycling reactions and the generation of reactive oxygen species (ROS) [5,6]. Wilson’s disease is associated with copper accumulation, displaying the signs of chronic liver cirrhosis, acute liver failure, neurologic symptoms, etc. [7,8,9]. Therefore, exquisite regulation of copper metabolism is crucial for normal development and health in humans and animals.

Copper homeostasis is closely regulated through uptake, storage, and export involving many key proteins, such as duodenal cytochrome b (DCYTB), copper transporter 1 (CTR1), copper chaperones, and copper-transporting P-type ATPases (ATP7A and ATP7B). DCYTB expression has been shown to maintain extracellular levels of ascorbate, which plays an important role in reducing from Cu (II) to Cu (I) [10]. CTR1, localizing on the apical membrane of the epithelial, delivers the Cu (I) from the intestinal lumen into cells [11,12]. Upon import, Cu (I) is chaperoned or sequestered. Additionally, ATP7A, located on the basolateral membrane, mediates Cu efflux out of the cells by binding and translocating 6 Cu (I) ions [10]. It is crucial for copper homeostasis to function properly, and aberrant expression or activity could result in serious disorders of copper metabolism. It was reported that *Ctr1* silencing in intestinal epithelial cells induced systemic copper deficiency resulting in death prior to weaning and ataxia in mice [13]. Moreover, a previous study revealed that *Ctr1* homozygous knockout led to early embryonic lethality in mice [14]. ATP7B mutation-induced hepatic copper accumulation (Wilson’s disease) results in liver cirrhosis, neurodegeneration, and anemia, mainly by exacerbating Fenton and Haber–Weiss reactions that produce hydroxyl radicals [15,16]. Alternatively, Menkes disease is caused by mutations in the copper transporter ATP7A, which results in a severe copper deficiency that can lead to death [17]. Therefore, a delicate regulation of the copper-transporter proteins is critical in maintaining copper homeostasis in the body. 

Increasing studies document that internal or external environmental stress could affect the absorption and metabolism of trace elements [18]. It is reported that psychological stress results in iron homeostasis disorder by disturbing intestinal iron absorption and tissue iron distribution, including the liver, intestine, spleen, hippocampus, cerebral cortex, and striatum [19,20,21]. In our previous studies, we found chronic stress resulted in hepatic iron accumulation [22,23,24], but long-term dexamethasone exposure decreased hepatic iron storage [25]. However, to our knowledge, relevant studies involving the relationship between stress and copper metabolism have been rare. Corticosterone (CORT), the major stress hormone produced in the cortex of the adrenal gland, plays a vital role in stress-induced HPA axis activity in rodents. Corticosterone (CORT) treatment was applied to develop the stress model by drinking water, injection, pellet implantation, etc. Oral CORT treatment via drinking water was thought to be an economical, safe, and non-invasive method to create a chronic stress model [26,27]. Here we use a CORT exposure model to uncover the relationship between chronic stress and copper metabolism and to investigate the underlying mechanism. 

## 2. Materials and Methods

### 2.1. Animals and Treatment

Fifty male C57BL/6J mice (6~8-week-old) purchased from the Comparative Medicine Centre of Yangzhou University were housed at constant temperature (20~26 °C) and humidity (40%~70%) with a light–dark cycle of 12 h at the Nanjing Agricultural University. After the one-week adaptation period, mice were allocated into the Control group (CON, drink 1% ethanol) and the Corticosterone group (CORT, drink 1% ethanol containing 100 μg/mL corticosterone) [28,29], respectively. Mice were subjected to free access to food and water, and recording of weight and feed intake every three days during the 28-day trial. At the end of the trial, mice were sacrificed under 25% urethane anesthesia to obtain blood, duodenum, and liver samples. Standard protocol for the treatment of mice by the Animal Ethics Committee of Nanjing Agricultural University (ethical review number: NJAU.No20210629098) was followed. 

### 2.2. Determination of Plasma Copper Content

The content of plasma copper was detected using the Copper Assay Kit (E010-1-1, Nanjing Jiancheng Corp., Nanjing, China) according to the instructions. Briefly, under acidic conditions, Cu (II) dissociates from ceruloplasmin and albumin. Ascorbic acid reduces Cu (II) to Cu (I), which reacts with complexing agent 3, 5-dibromo-PAESA to produce a blue complex. OD value at 600 nm is collected by a full-automatic microplate reader. 

### 2.3. Determination of Liver Copper Content

The samples of the liver were digested following the previously described method [25]. Then, the digested samples were determined to calculate the tissues’ copper content using an atomic absorption spectrophotometer (ICE3500, Thermo Scientific, Wilmington, DE, USA).

### 2.4. Determination of Plasma and Liver Ceruloplasmin and Cu/Zn-SOD Activity

The content of ceruloplasmin and Cu/Zn-SOD activity was detected using the ceruloplasmin assay kit (A029-1-1, Nanjing Jiancheng Corp., Nanjing, China) and Cu/Zn superoxide dismutase (Cu/Zn-SOD) assay kit (A001-2-1, Nanjing Jiancheng Corp., Nanjing, China) according to the supplier’s instructions.

### 2.5. Histological Evaluation

*H&E* staining for the duodenum and liver were performed to determine the extent of tissue damage as previously described [24]. Briefly, duodenum and liver tissues were sectioned, paraffin-embedded, dewaxed, rehydrated and stained with *H&E*. The immunohistochemistry was performed according to the instructions of the kit (I003-1-1, Nanjing Jiancheng Corp., Nanjing, China). The liver paraffin sections were baked at 60 °C, then soaked in xylene to deparaffinize, and gradient ethanol immersion rehydrated the sections. Then the tissue sections were heated in a water bath in 0.01 M citrate buffer (pH 6.0) to restore the antigen. An amount of 0.2% TritonX-100 was used to make the tissues permeable, then treated for 20 min with 3% hydrogen peroxide to inactivate endogenous peroxidase, then washed 3 times with PBS containing 0.05% Tween-20 to mask non-specific binding sites. The tissues were incubated with 10% goat serum-blocking solution for 2 h. MT antibody (DF6755, Affinity, Cincinnati, OH, USA, dilution, 1:100) was incubated overnight at 4 °C, and biotinylated secondary antibodies were incubated for 30 min at 37 °C. Signals were amplified with SABC for 30 min. The slices were incubated with DBA chromogenic solution at room temperature for 5~10 min, stained with hematoxylin, dehydrated, and the sheet was sealed with neutral resin. 

### 2.6. Isolation of Primary Hepatocytes and Assessment of Reactive Oxygen Species (ROS) 

Primary murine hepatocytes were extracted as described before [30]. Primary hepatocytes from the control and corticosterone-treated mice were isolated by perfusion with collagenase type IV (mbs165, Nanjing Jiancheng Corp., Nanjing, China). In brief, mice were anesthetized with an intraperitoneal injection of 25% urethan, and the abdomen was opened and perfused with 20 mL PBS via the left atrium of the heart to remove excess red blood cells in the liver. Subsequently, minced livers were digested with 1 mg/mL of collagenase type IV in PBS for 30 min at 37 °C, then filtered with a 200-mesh cell sieve and centrifuged to obtain cell precipitation.

To measure intracellular ROS generation in mouse primary hepatocytes, we used a ROS assay kit from Beyotime Biotechnology (S0033, Shanghai, China). Each sample was incubated for 30 min at 37 °C with a fluorescent probe (10 μM). The fluorescence intensity in the FITC channel was monitored by flow cytometry.

### 2.7. Total RNA Isolation and Real-Time PCR

In brief, total RNA was isolated from duodenum, liver, and Caco-2 samples using TRIzol Reagent (15596018, Tsingke Biotechnology Co., Nanjing, China), then its quantity and quality were determined using a Nano Drop-1000 (Thermo Scientific, Wilmington, DE, USA). Reverse transcription was performed using HiScript II Q RT SuperMix for qPCR (R223-01, Vazyme Biotech Co., Shanghai, China) on the QuantStudio 6 Flex Real-Time PCR system by Applied Biosystems (Applied Biosystems, Waltham, MA, USA). The data were calculated with the 2^−ΔΔCt^ method using tubulin-β as the reference gene. The primers used were derived from the coding region of each gene and are listed in Supplementary Table S1.

### 2.8. Protein Extraction and Western Blotting Analysis

Protein was extracted from duodenum, liver, Caco-2 and HepG2 cell samples using the lysis RIPA buffer as previously described [31]. An Easy Ⅱ Protein Quantitative Kit (DQ111, TransGen Biotech., Beijing, China) was used to detect the protein concentration. Protein samples were separated by SDS-PAGE on a 6%~12% gel and transferred to nitrocellulose membranes. After incubating with the primary antibody overnight at 4 °C, the slides were incubated with the secondary antibody for 2 h at room temperature. In Image J (NIH Image J system, Bethesda, MD, USA), band density analysis was performed using images captured by the VersaDoc system (Bio-Rad, Hercules, CA, USA). The density of each protein band was normalized by that of Tubulin-α or Actin, the internal control. Details of primary and secondary antibodies are listed in Supplementary Table S2. In addition, the original images for blots and gels are shown in the Supplementary Materials.

### 2.9. Cell Culture and Immunofluorescence

Freshly thawed Caco-2 cells or HepG2 cells were cultured in DMEM + 10% fetal bovine serum medium at 37 °C at 5% CO_2_. When cell confluence reached 80%, cells were subcultured in a 6-well/24-well plate and incubated for 24 h, then the cells were treated with the following reagents: Corticosterone (1 μM or 10 μM, ab143597, Abcam, MA, USA), and/or RU486 (10 μM, M8046, Sigma-Aldrich, St. Louis, MO, USA), a GR antagonist. After 12 h or 24 h of treatment, the cells were collected to extract RNA and protein or immunofluorescence.

Caco-2 cells or HepG2 cells were fixed in 4% neutral paraformaldehyde for 20 min in a shaker. PBS containing 0.5% TritonX-100 was added to break the membrane at room temperature for 20 min, blocked with 5% FBS, and then incubated with rabbit anti-GR antibody (24050-1-AP, Proteintech., Chicago, IL, USA) overnight at 4 °C and goat anti-rabbit IgG (ab150077, Abcam, MA, USA) for 1 h. DAPI (DAPI dilactate, Cat #D3571, Invitrogen, CA, USA) was used to stain the cell nuclei. Subsequently, the samples were viewed with a fluorescence microscope (Leica, DMI6000 B, Germany).

### 2.10. Chromatin Immunoprecipitation (ChIP) Assay

ChIP was carried out as previously described [32]. Briefly, Caco-2 cells were washed twice with pre-chilled PBS containing protease inhibitors (Roche, Basel, Switzerland), cross-linked in 1% formaldehyde, then the reaction was terminated with 2.5 mol/L glycines. Next, the chromatin was sonicated to an average length of about 200~500 bp, and then the protein-DNA complex was incubated with 4 μg GR antibody (24050-1-AP, Proteintech., Chicago, IL, USA) or no antibody overnight at 4 °C. Subsequently, the immunoprecipitated chromatin complexes were captured with Protein G Sepharose beads (sc-2003, Santa Cruz, California, USA). The samples were then reverse cross-linked in a 65 °C water bath for 1 h to release DNA from the immunoprecipitated complex and purified DNA was used as a template for qPCR. JASPAR 2020 (http://jaspar.genereg.net. Accessed on 10 January 2020) was used to predict the putative GRE in the *Ctr1* promoter. The primers used to amplify the sequences covering these putative GREs are listed in Supplementary Table S3.

### 2.11. Luciferase Reporter Assay

According to the result of ChIP-qPCR, the GRE site was identified. With the GRE site as the center, the double luciferase PGL3-WT plasmid was constructed with 450 bp, and the GRE site was mutated to construct the PGL-MUT plasmid, only the first half 200 bp without the GRE site was retained as the PGL3-200 plasmid. Caco-2 cells were plated in 24-well plates, and 0.5 μg double luciferase reporter plasmid was transfected with transfection reagent jetPRIME (114-15, Polyplus Transfection, France) when the cells reached 60~70% confluence. Cells were lysed and detected on the GlOMAXTM 96 microplate luminometer using the dual-luciferase reporter assay system (11402ES60, Yepsen, Shanghai, China), according to the manufacturer’s instructions at 24 h post-transfection. 

### 2.12. Statistical Analysis

Differences between groups were analyzed by two-tailed Student’s *t*-test and one-way ANOVA using SPSS 20.0 software (SPSS Inc., Chicago, IL, USA). Data are expressed as means ± SEM. There was a statistically significant difference if *p* < 0.05. 

## 3. Results

### 3.1. Chronic Corticosterone Exposure Markedly Increases Average Daily Food Intake and Final Body Weight in Mice

**Figure 1:**
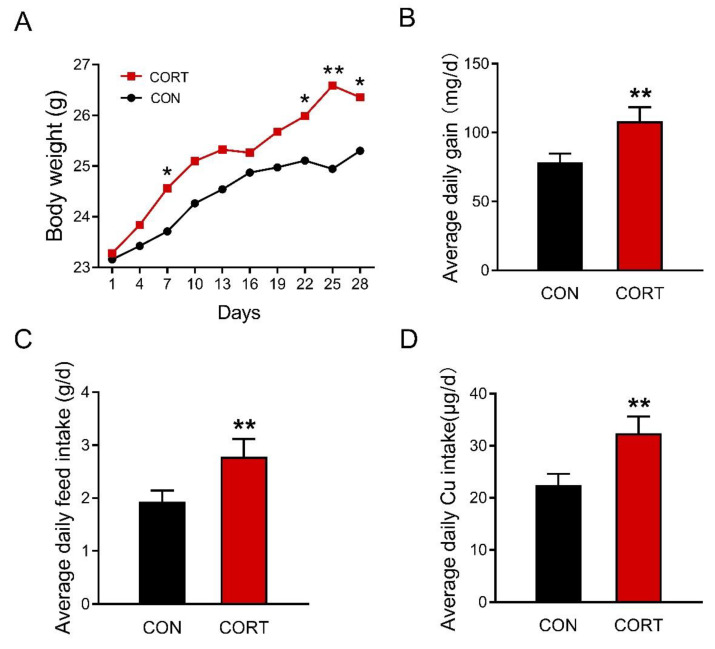
Corticosterone exposure increases average daily food intake and final body weight in mice. (**A**) The change of mouse body weight over time during the experiment (n = 25). (**B**) Average daily feed intake (n = 5). (**C**) Average daily copper intake (n = 5). (**D**) Average daily copper intake (n = 5). CON = control group; CORT = corticosterone exposure group. Values are means  ±  SEM, * *p*  <  0.05, ** *p*  <  0.01.

Chronic corticosterone exposure significantly increased the body weight of mice on day 7 and day 22 to day 28 during the 28-day trial (Figure 1A, *p* < 0.05 or *p* < 0.01). The average daily gain (Figure 1B, *p* < 0.01), average daily feed intake (Figure 1C, *p* < 0.01), and average copper intake (Figure 1D, *p* < 0.01) were also markedly enhanced in mice exposed to chronic corticosterone treatment. Meanwhile, it was found that chronic corticosterone exposure greatly raised the plasma *p* < 0.01 corticosterone level in mice, indicating that the stress model was successfully established (Figure S1, *p* < 0.01).

### 3.2. Chronic Corticosterone Exposure Significantly Reduces the Plasma and Hepatic Copper-Related Parameters in Mice

As shown in Table 1, chronic corticosterone exposure disrupted the copper metabolism of mice, which manifested as a significant decrease in the plasma copper level (*p* < 0.05), hepatic copper content (*p* < 0.01), plasma ceruloplasmin activity (*p* < 0.01), and plasma and hepatic Cu/Zn-SOD activity (*p* < 0.05 or *p* < 0.01).

### 3.3. Chronic Corticosterone Exposure Damages the Duodenal Structure and Impairs the Expression of Copper Transport-Related Genes in Mice

**Figure 2:**
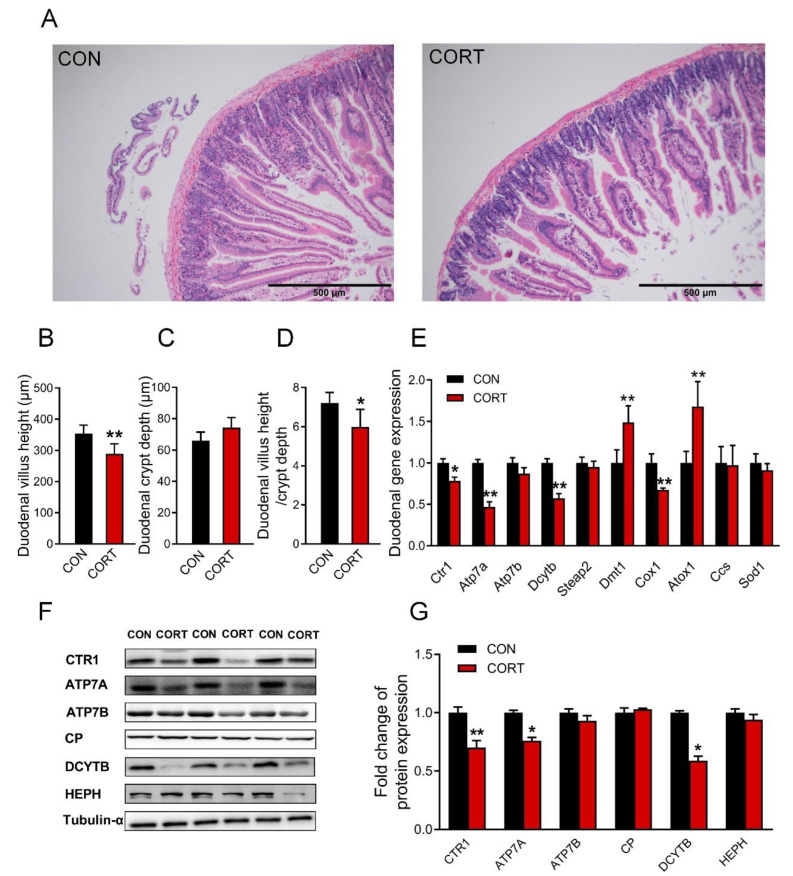
Corticosterone exposure damages the structure of the duodenum and reduces the content of copper absorption and transport-related proteins. (**A**) *H&E* staining of the duodenum (n = 3)**.** (**B**–**D**) Duodenal villi height, crypt depth, and the ratio of the two groups (n = 12). (**E**) mRNA expression of *Ctr1*, *Atp7a*, *Atp7b*, *Dcytb*, *Steap2*, *Dmt1*, *Cox1*, *Atox1*, *Ccs*, and *Sod1* were evaluated by qPCR (n = 6). (**F**,**G**) Western blot analyses of CTR1, ATP7A, ATP7B, CP, DCYTB, HEPH and Tubulin-α proteins (n = 6). CON = control group; CORT = corticosterone exposure group. Values are means ± SEM, * *p* < 0.05, ** *p* < 0.01.

Chronic corticosterone exposure induced damage to the duodenal morphology in mice, which manifested as a significant shortening of intestinal villi (Figure 2A,B, *p* < 0.01) and an extremely decreased ratio of the villus height to crypt depth (Figure 2D, *p* < 0.05); duodenal crypt depth had no significant effect (Figure 2C, *p* > 0.05). The results of real-time PCR showed that the duodenal *Ctr1* (*p* < 0.05), *Atp7a* (*p* < 0.01), *Dcytb* (*p* < 0.01), and *Cox1* (*p* < 0.01) genes were greatly decreased, while the *Dmt1* (*p* < 0.01) and *Atox1* (*p* < 0.01) genes were greatly increased (Figure 2E) in mice exposed to corticosterone. Meanwhile, the expression of duodenal CTR1 (*p* < 0.01), ATP7A (*p* < 0.05), and DCYTB (*p* < 0.05) proteins were markedly reduced in mice exposed to corticosterone (Figure 2F,G). In addition, the original images for blots and gels are shown in the Supplementary Materials.

### 3.4. Chronic Corticosterone Exposure Induces Hepatic Damage and Disrupts Hepatic Copper Metabolism in Mice

**Figure 3:**
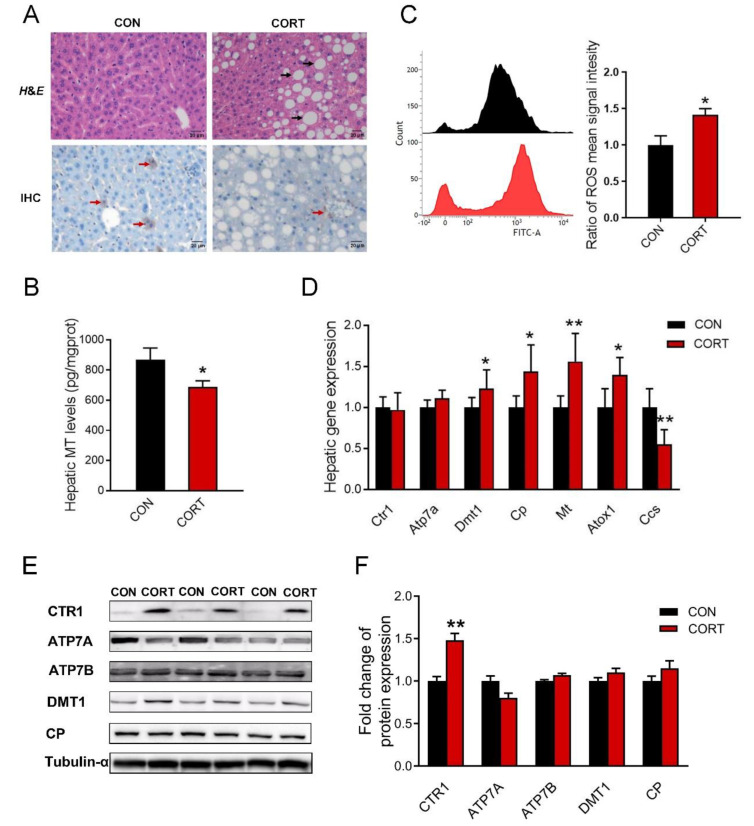
Chronic corticosterone exposure induces hepatic damage and disrupts hepatic copper metabolism in mice. (**A**) *H&E* staining of mouse liver and immunohistochemistry of metallothionein (n = 3); black arrow: lipid droplets; red arrow: metallothionein position. (**B**) The content of metallothionein in mouse liver detected by ELISA kit (n = 8). (**C**) Intracellular ROS levels were measured using flow cytometry (n = 3). (**D**) mRNA expression of *Ctr1*, *Atp7a*, *Dmt1*, *Cp*, *Mt*, *Atox1* and *Ccs* were evaluated by qPCR (n = 6). (**E**,**F**) Western blot analyses of CTR1, ATP7A, ATP7B, DMT1, CP and Tubulin-α proteins (n = 6). CON = control group; CORT = corticosterone exposure group. Values are means ± SEM, * *p* < 0.05, ** *p* < 0.01.

*H&E* staining results showed that chronic corticosterone exposure induced droplet deposition in the liver of mice (Figure 3A, up). The immunohistochemical detection (Figure 3A, down) and ELISA test (Figure 3B, *p* < 0.05) exhibited that chronic corticosterone exposure markedly down-regulated the metallothionein level in mice. Moreover, corticosterone induced hepatic oxidative stress via enhancing hepatic ROS production (Figure 3C, *p* < 0.05) in mice. In addition, the results of real-time PCR showed that the hepatic expression of *Dmt1* (*p* < 0.05), *Cp* (*p* < 0.05), *Mt* (*p* < 0.01), and *Atox1* (*p* < 0.05) were significantly increased, while the *Ccs* (*p* < 0.01) mRNA expression was greatly decreased in mice exposed to chronic corticosterone treatment (Figure 3D). As shown in Figure 3E,F, chronic corticosterone exposure greatly increased the hepatic CTR1 protein expression in mice (*p* < 0.01). In addition, the original images for blots and gels are shown in the Supplementary Materials.

### 3.5. Corticosterone Down-Regulates CTR1 Protein Expression in Caco-2 Cells and HepG2 Cells

**Figure 4:**
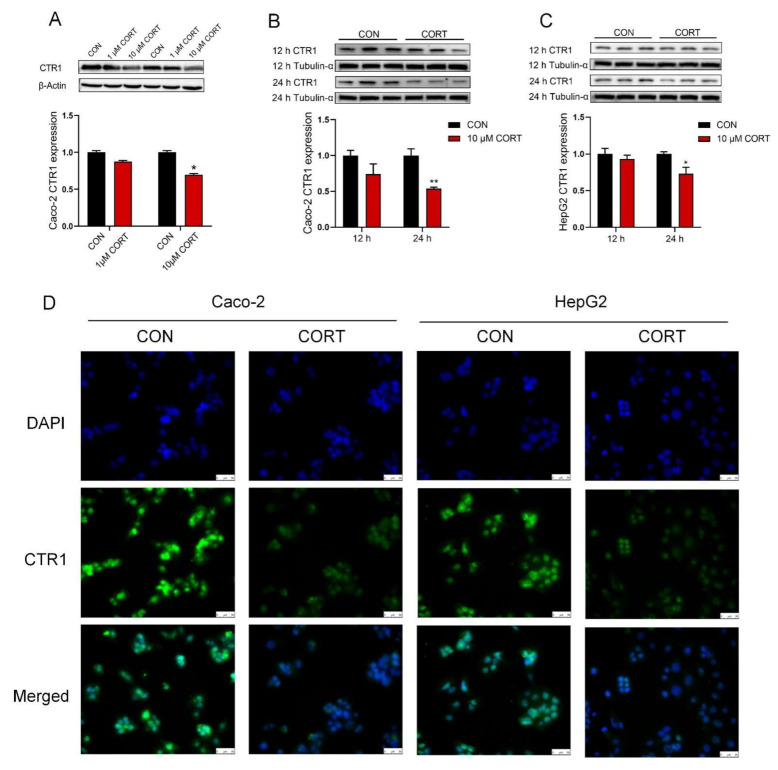
Corticosterone down-regulates CTR1 protein levels on Caco-2 and HepG2 cells. (**A**) The expression of CTR1 protein was analyzed by Western blot of Caco-2 cells treated with different concentrations of corticosterone (CORT, 1 μM and 10 μM) for 24 h. (**B**) Western blot analysis of CTR1 protein expression in Caco-2 cells with corticosterone (CORT, 10 μM) treatment for 12 or 24 h. (**C**) Western blot analysis of CTR1 protein expression in HepG2 cells with corticosterone (CORT, 10 μM) treatment for 12 or 24 h. (**D**) Immunofluorescence analysis of CTR1 protein expression in Caco-2 and HepG2 cells with corticosterone (CORT, 10 μM) treatment for 24 h. n = 3, CON = control group; CORT = corticosterone exposure group. Values are means ± SEM, * *p* < 0.05, ** *p* < 0.01.

CTR1 protein expression was similarly down-regulated in Caco-2 cells (Figure 4A,B, *p* < 0.01 or *p* < 0.05) and HepG2 cells (Figure 4C, *p* < 0.05) exposed at a concentration of 10 µM corticosterone for 24 h, but not for 12 h. Immunofluorescence staining results also revealed that the expression of CTR1 was greatly lowered in Caco-2 cells and HepG2 cells upon corticosterone exposure for 24 h (Figure 4D). In addition, the original images for blots and gels are shown in the Supplementary Materials.

### 3.6. Corticosterone Inhibits CTR1 Protein Expression Accompanied by an Increase in Nuclear GR Protein in Caco-2 Cells

**Figure 5:**
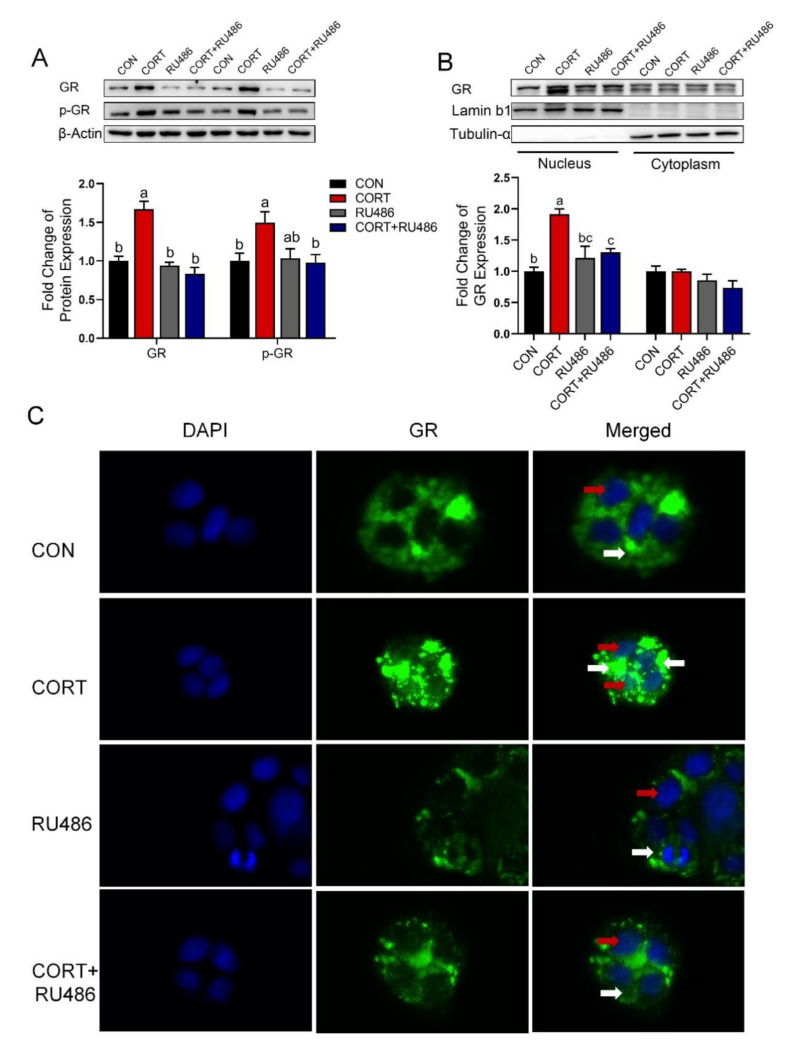
Corticosterone activates GR and promotes its entry into the nucleus in Caco-2 cells. (**A**) Western blot analysis of GR and p-GR protein expression in Caco-2 cells with corticosterone (CORT, 10 μM) treatment in the presence and absence of GR antagonist RU486 (10 μM) for 24 h. (**B**) Western blot analysis of GR protein expression in nucleus and cytoplasm with corticosterone (CORT, 10 μM) treatment in the presence and absence of GR antagonist RU486 (10 μM) for 24 h. (**C**) Immunofluorescence analysis of GR protein expression and localization in Caco-2 cells with corticosterone (CORT, 10 μM) treatment in the presence and absence of GR antagonist RU486 (10 μM) for 24 h. n = 3, CON = control group; CORT = 10 μM corticosterone; RU486 = 10 μM mifepristone; CORT + RU486 = 10 μM corticosterone + 10 μM mifepristone. Values are means ± SEM. Different lowercase letters represent significant (*p* < 0.05). Red arrow: distribution of GR in the nucleus. White arrow: distribution of GR in the cytoplasm.

Corticosterone exposure activated glucocorticoid receptor (GR) through increasing expression of total GR protein (*p* < 0.01) and phosphorylated GR (p-GR, *p* < 0.05) protein in Caco-2 cells (Figure 5A). Western blotting results showed that corticosterone greatly up-regulated nuclear GR expression, but GR antagonist mifepristone (RU486) exhibited a partial antagonistic effect on GR in Caco-2 cells (Figure 5B, *p* < 0.05 or *p* < 0.01). Additionally, the results of immunofluorescence also exhibited that corticosterone exposure markedly increased the translocation of GR from the cytosol into the nucleus, whereas RU486 reversed this change in Caco-2 cells (Figure 5C). In addition, the original images for blots and gels are shown in the Supplementary Materials.

### 3.7. GR Binding to GRE in the Promoter of the Ctr1 Gene to Reduce Its Protein Expression

**Figure 6:**
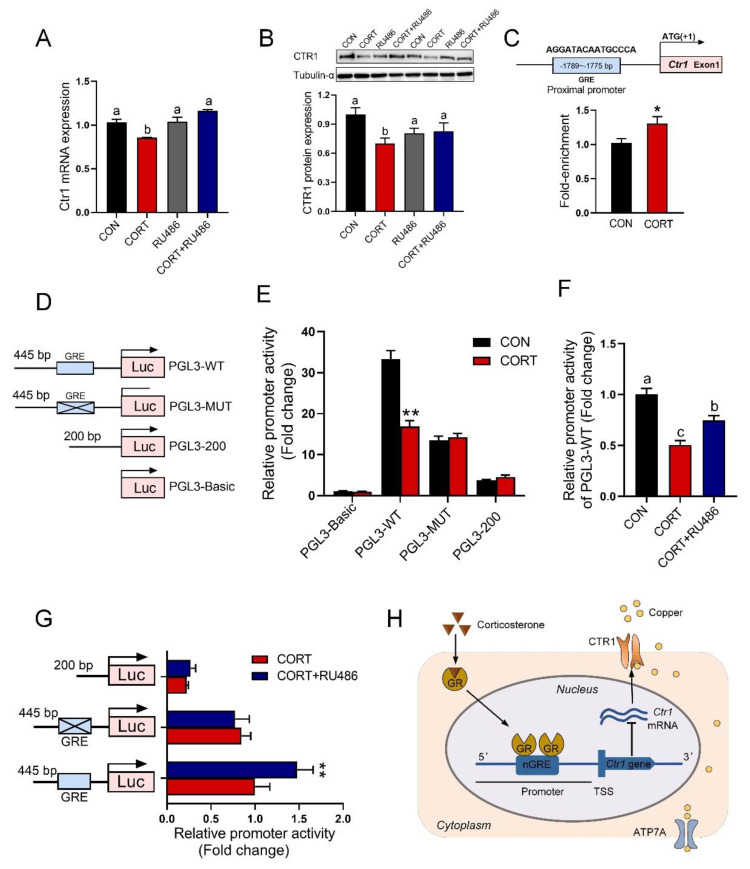
GR binding to the promoter of the *Ctr1* gene reduces its protein expression. (**A**,**B**) mRNA expression and protein content of CTR1 in Caco-2 cells with corticosterone (CORT, 10 μM) treatment in the presence and absence of GR antagonist RU486 (10 μM) for 24 h (n = 3). (**C**) Schematic representation of GR transcription factor binding promoter of *Ctr1* (up) and ChIP-PCR assay was used to measure the binding of GR on *Ctr1* promoter in the Caco-2 cells after 24 h treatment with 10 μM CORT (down, n = 3). (**D**) Schematic representation of truncated or mutated *Ctr1* pGL3-plasmids. (**E**) The promoter activity of truncated or mutated *Ctr1* pGL3-plasmids in the absence or presence of 10 μM CORT stimulated for 24 h (n = 4). (**F**) The promoter activity of *Ctr1* pGL3-WT in the absence of 10 μM CORT stimulated with/without 10 μM RU486 for 24 h (n = 4). (**G**) The promoter activity of *Ctr1* pGL3-WT plasmids, pGL3-MUT (site-directed mutation GR binding site) and pGL3-200 plasmids (lacking the GRE region) in the absence of 10 μM CORT stimulated with/without 10 μM RU486 for 24 h (n = 4)**.** (**H**) Graphical abstract. Corticosterone activates glucocorticoid receptors, leading to increased entry of GR into the nucleus. GR entering the nucleus binds to the nGRE site in the promoter region of *Ctr1*, inhibits *Ctr1* transcription and reduces the expression of CTR1 protein, which is partly responsible for the inhibition of copper absorption. CON = control group; CORT = 10 μM corticosterone; RU486 = 10 μM mifepristone; CORT + RU486 = 10 μM corticosterone + 10 μM mifepristone. Values are means ± SEM, * *p* < 0.05, ** *p* < 0.01. Different lowercase letters represent significance (*p* < 0.05).

Corticosterone greatly impaired the expression of CTR1 at the mRNA (Figure 6A, *p* < 0.05) and protein levels (Figure 6B, *p* < 0.05), whereas RU486 reversed this change in Caco-2 cells. The bioinformatics analysis revealed the glucocorticoid regulatory element (GRE) in the promoter regions of the human *Ctr1* gene (Figure 6C, up). The ChIP-PCR analysis showed that GR bound specifically to the promoter of *Ctr1*, and exhibited a higher level of GR binding to the *Ctr1* gene in Caco-2 cells treated with corticosterone (Figure 6C, down, *p* < 0.05). In order to verify the influence of GRE on the *Ctr1* gene promoter, different DNA fragments were cloned into pGL3-basic plasmid, and fused to a luciferase reporter gene (Figure 6D). As shown in Figure 6E, the DNA fragment containing GRE (PGL3-WT) showed the lower promoter activity (*p* < 0.01), but deletion or mutation of GRE in the DNA fragment did not differ the promoter activity in Caco-2 cells upon corticosterone exposure. In addition, we found that RU486 disrupted the binding of GR to GRE, resulting in a major increase of the promoter activity in Caco-2 cells treated with corticosterone (Figure 6F,G, *p* < 0.01). See Figure 6H for a graphical abstract.

## 4. Discussion

Chronic exposure to GCs, especially at high doses, is likely to contribute to increasing food intake and body weight in both humans and rodents [33,34,35,36,37]. It is documented that mice exposed to high levels of CORT (100 μg/mL) result in dramatic increases in body weight and food intake and triglyceride levels, but a lower dose of CORT (25 μg/mL) had no effect [34]. Harno et al. reported that CORT treatment (75 μg/mL in drinking water) resulted in weight gain over the 3-week experimental period in mice [38]. Similar to previous studies, a 4-week treatment of high CORT (100 μg/mL in drinking water) increased the body weight and food intake of mice in the current study. However, low doses of CORT (10~40 mg/kg) or dexamethasone (0.3 μg/mL) resulted in weight loss [39,40]. These differences in body weight and food intake induced by glucocorticoid are dose-dependent. High glucocorticoid levels and enhanced food intake could partly explain the glucocorticoid-induced body weight gain.

Mice suffering chronic corticosterone exposure exhibit copper deficiency, reductions in plasma copper level, ceruloplasmin activity, and copper-requiring enzyme Cu/Zn-SOD activity, as well as decreased hepatic copper content, metallothionein content and Cu/Zn-SOD activity in mice. It has been demonstrated that the majority of copper in plasma is stored in ceruloplasmin, whose function and metabolism depend on copper availability [41,42]. Hepatic metallothionein binds copper with high affinity, suggesting a role in regulating the bioavailability of copper [43,44]. In addition, similar to other studies, chronic restraint stress exhibited severe villous epithelial atrophy, increases in crypt depth, and inducement of hepatic oxidative stress and hepatic steatosis in mice [45,46]. Concordantly, chronic corticosterone exposure significantly down-regulated the expression level of duodenal CTR1, ATP7A, and DCYTB proteins, which was strongly related to the control of copper absorption in mice. CTR1 is localized to the apical membrane of intestinal epithelial cells and functions for intestinal dietary copper uptake [13,47]. It is noted that *Ctr1*^int/int^ mice exhibited dramatically lower circulating copper levels [13], whereas upregulation of CTR1 is known to promote copper uptake in vitro [48]. In the current study, corticosterone treatment markedly down-regulated the CTR1 protein expression in Caco-2 cells and HepG2 cells at 24 h, but up-regulated the hepatic CTR1 expression in mice. Chronic corticosterone exposure probably activates some post-transcriptional regulatory or multiple regulatory mechanisms to balance the copper concentration in the mouse liver. In this study, down-regulated intestinal CTR1 expression is partly responsible for lowered circulating copper levels in mice treated with chronic corticosterone exposure. Chronic corticosterone exposure impairs the CTR1 expression at mRNA and protein levels, indicating that CTR1 expression is regulated at the level of transcription.

Circulating GCs enter the cells via diffusion across the cell membrane and interact with GR. Translocating from the cytoplasm into the nucleus, the glucocorticoid response element (GRE) or other transcription factors are bound by the glucocorticoid-GR complex upon hormone binding to regulate gene transcription via the DNA-binding mechanism [49,50] or protein–protein cross-talk mechanism [51,52]. In a few cases, the binding of GR to negative GREs (nGREs) at promoter regions has been implicated in GC-induced transrepression. It has been demonstrated that glucocorticoid-induced inhibition of thymic stromal lymphopoietin (TSLP) transcription is mediated through GR and corepressors binding to a negative GRE located in the TSLP promoter region [49]. He et al. also reported that dexamethasone, also known as glucocorticoids, down-regulated SLC7A5 expression via GR bound directly to negative GRE at the proximal promoter sequence [50]. In our study, a bioinformatics analysis of 2 kb of DNA located in the upstream promoter region revealed the GRE binding motif in human *Ctr1* genes. The *Ctr1* gene containing GRE motif in Caco-2 cells was analyzed for (1) repression by CORT-treatment, (2) prevention of this repression by RU486 cotreatment, (3) repressing activity of their nGREs in vitro, which taken altogether represent the signature of GRE-mediated transrepression. Upon CORT-treatment of Caco-2 cells, we found that the inhibition effect of CORT on *Ctr1* gene expression and nuclear GR translocation [53] could be prevented by excess RU486 co-treatment. Moreover, the ChIP assay showed that GR binding allowed confirmation of the predicted GRE in the promoter region of *Ctr1* which was bound by GR in vitro [54]. In addition, pGL3-based luciferase plasmids containing *Ctr1* promoter (expressing full-length GRE or mutated GRE), were used to study whether GRE could function as efficient negative GRE [55,56]. In our study, transient transfection-based reporter assays showed that GRE mutation or deletion interfered with GR-*Ctr1* interactions representing an unaffected promoter–reporter activity in Caco-2 cells treated with CORT. Additionally, blocking the GR signal pathway by GR antagonist RU486 attenuated CORT-induced reduction in promoter–reporter activity and expression of *Ctr1* mRNA in Caco-2 cells. Therefore, we show that induction of the *Ctr1* promoter by GR would inhibit CTR1 protein expression via a negative GRE-dependent pathway. 

## 5. Conclusions

Taken together, CORT impairs copper transport by down-regulating intestinal CTR1 expression via GR-mediated transcriptional inhibition. These results provide a novel insight into intestinal transport inhibition of copper induced by chronic stress.

## Figures and Tables

**Table 1:** Corticosterone exposure disturbs plasma and hepatic copper-metabolism-related parameters in mice.

Parameters	Treatments		*p*-Value
	CON	CORT	
**Plasma**			
Cu level (μmol/L)	28.92 ± 1.23	25.34 ± 2.14	0.03
Ceruloplasmin activity (U/L)	19.44 ± 1.80	12.11 ± 1.41	0.00
Cu/Zn-SOD (U/mL)	104.13 ± 3.70	87.61 ± 5.97	0.03
**Liver**			
Cu content (μg/g)	6.88 ± 0.21	4.51 ± 0.26	0.00
Ceruloplasmin activity (U/mg protein)	8.40 ± 0.92	9.50 ± 1.00	0.43
Cu/Zn-SOD (U/mg protein)	277.47 ± 4.24	251.99 ± 6.55	0.00

Note: CON = control group; CORT = corticosterone exposure group. Values were expressed as means ± SEM, n = 8, and *p* < 0.05 means a significant difference between the two groups.

## Data Availability

Not applicable.

## Supplementary Materials

The following supporting information can be downloaded at: https://www.mdpi.com/article/10.3390/biology12020197/s1, Table S1, Nucleotide sequences of specific primers for qPCR; Table S2, Details of antibodies used in the experiment; Table S3, Nucleotide sequences of specific primers for CHIP-PCR; Figure S1, Corticosterone-exposed mice plasma corticosterone levels are up-regulated. In addition, see the original images for blots and gels in the Supplemental Materials.
