# Supplementary material for: Chronic Corticosterone Exposure Suppresses Copper Transport through GR-Mediated Intestinal CTR1 Pathway in Mice
Source: Biology (Basel). 2023 Jan 28;12(2):197. doi: 10.3390/biology12020197 (PMC9953443; doi:10.3390/biology12020197)
Supplement: Supplementary file 1 [file biology-12-00197-s001.zip › biology-2120308-SI.pdf]

Supplementary Table S1. Nucleotide sequences of specific primers for qPCR

Target genes	GenBank No.	Sequences (5' to 3')
Atp7b	NC_000074.7	F：GGGGACGATGCCTGAACAG
		R：TAGCCAACATTGTCGAAGGCG
Atox1	NC_000077.7	F：CTGTTTTGTTGAGGGTTGCCA
		R：GGGAGGAGTGGAGTTCAACA
Ccs	NC_000085.7	F：CTCGCTCCTCCCAGATAGTG
		R：CAGCCTGGTTATCGATGAGGG
Cox	NC_000067.7	F：GAAGAAGCCACTGAAGCCCT
		R：CCTCATGCACTCCTTGTGGG
Steap2	NC_000071.7	F：ACCGCTTTTACACACCACCA
		R：TCCTCCCATGCCTTCGTCTA
Sod1	NC_000082.7	F：CCACTTCGAGCAGAAGGCAA
		R：AGTCACATTGCCCAGGTCTC
Dmt1	NC_000081.7	F：TCACCATCGCAGACACTTTTG
		R：GACAGGACGGCACGAACAT
Atp7a	NC_000086.8	F：CTCGCAAACAGCACAGGGA
		R：GTGGCTCTTTCATGTCTGCTG
Dcytb	NC_000068.8	F：CTGTCGGTGATCTTCGTGCT
		R：GATGAAGACGAAGCCGGTCA
Ctr1	NC_000070.7	F：TGCCGATGGTTTGGTGTTTG
		R：AACCATATCAAAGGCTCTGTGG
Mt	NC_000074.7	F：TCCTGCAAGAAGAGCTGCTG
		R：CTGTTCGTCACATCAGGCAC
Cp	NC_000069.7	F：CATCGTGAGGTGCCCTACAG
		R：CATCGTGAGGTGCCCTACAG
Tubulin-β	NC_000074.7	F：CCCATCTACGAGGGCTAT
		R：TGTCACGCACGATTTCC

Supplementary Table S2. Details of antibodies used in the experiment

Antibodies	Source	Catalogue no.	Dilution ratio
Primary antibody			
CTR1	Novus	NB100-402SS	1:200(IF)
CTR1	Affinity	DF13356	1:1000
ATP7A	Affinity	DF8506	1:1000
DMT1	Abcam	ab55735	1:1000
CP	Proteintech	21131-1-AP	1:1000
DCYTB	Proteintech	26735-1-AP	1:1000
ATP7B	Proteintech	19786-1-AP	1:1000
GR	Proteintech	24050-1-AP	1:200~1000
p-GR (Ser211)	Cell Signaling Technology	4161S	1:1000
HEPH	Proteintech	11148-1-AP	1:1000
MT	Affinity	DF6755	1:100
GAPDH	Bioworld	MB001H	1:10000
β-actin	Bioworld	bs6007M	1:10000
Tubulin-α	Bioworld	BS1699	1:10000
Secondary antibody			
Goat anti-Mouse IgG (H+L) -HRP	Bioworld	BS12478	1:10000
Goat anti-Rabbit IgG (H+L)-HRP	Bioworld	BS13278	1:10000
Rabbit Anti-Goat IgG (H+L)-HRP	Bioworld	BS30503	1:10000
Goat Anti-Rabbit IgG H&L(Alexa Fluor® 488)	Abcam	ab150077	1:1000

Supplementary Table S3. Nucleotide sequences of specific primers for CHIP-PCR

Target genes	GenBank No.	Sequences (5’ to 3’)
CTR1	NC_000070.7	F：AGGCCCCATACACAGGCATA
		R：GAATGCCTGCACCCACTTAG

Figure captions

Figure S1. Corticosterone-exposed mice plasma corticosterone levels are up-regulated.

Figure S1

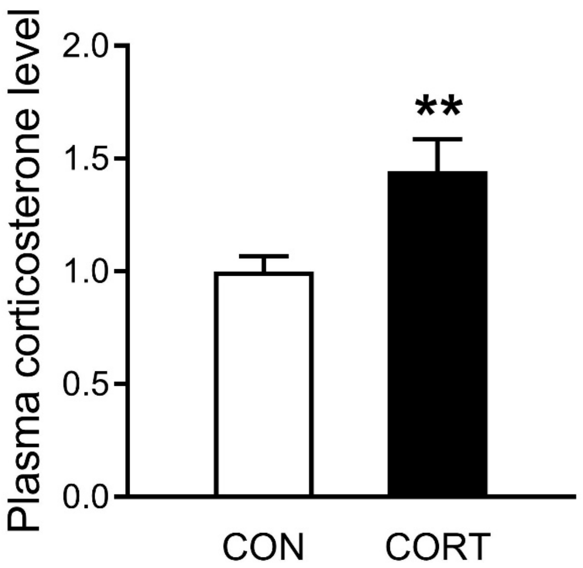

The following supplementary files are the images of full blots in protein analysis and H&E staining. The molecular weight markers have been labeled.

Figure S2A Mouse duodenum H&E staining, the first three are the control group, the last three are the corticosterone group, n=3

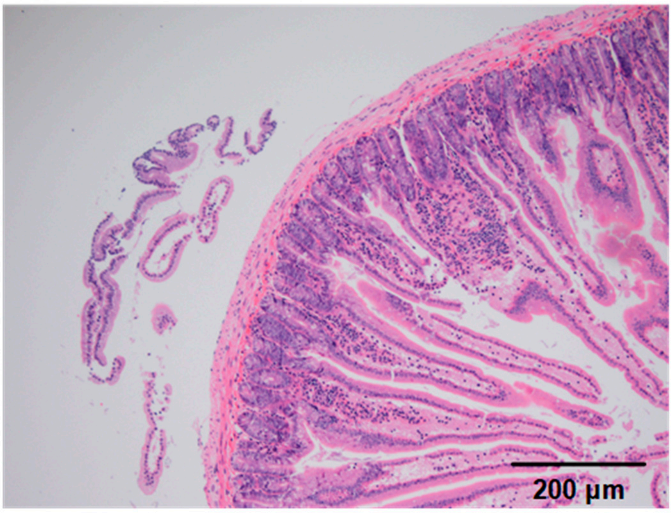

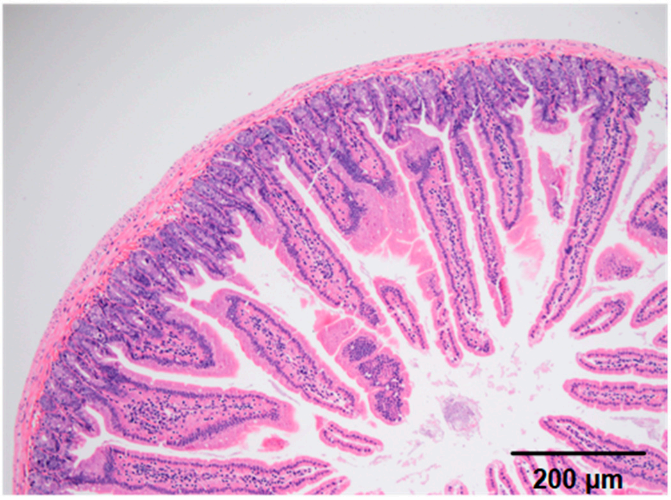

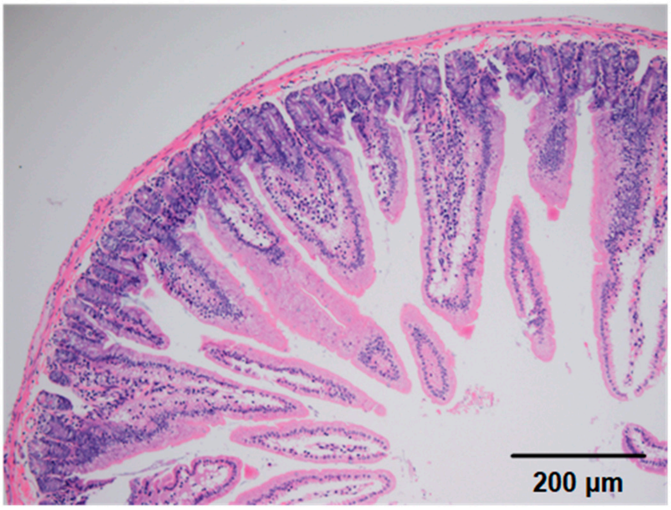

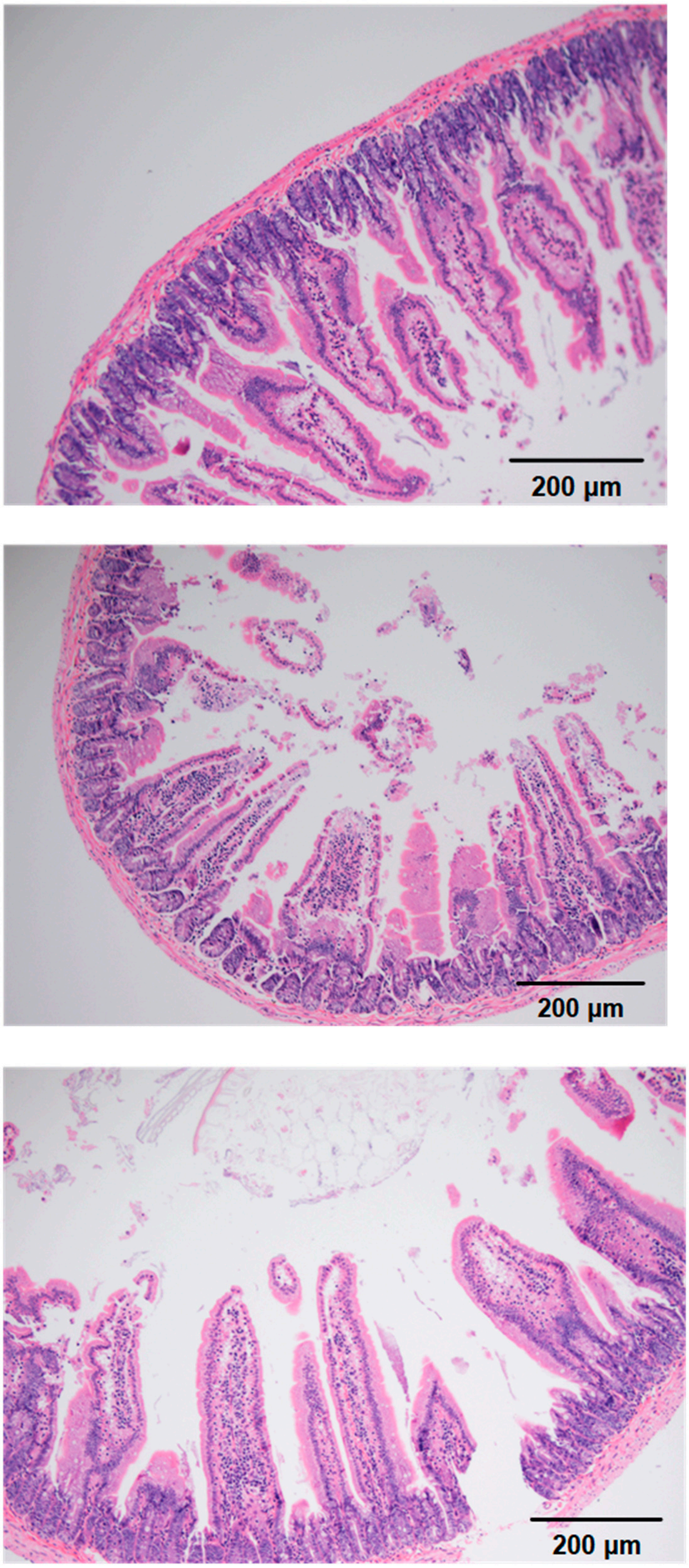

Figure S2F CTR1, ATP7A, ATP7B, CP, DCYTB,HEPH,Tublin-α bands observed in duodenum r of mice

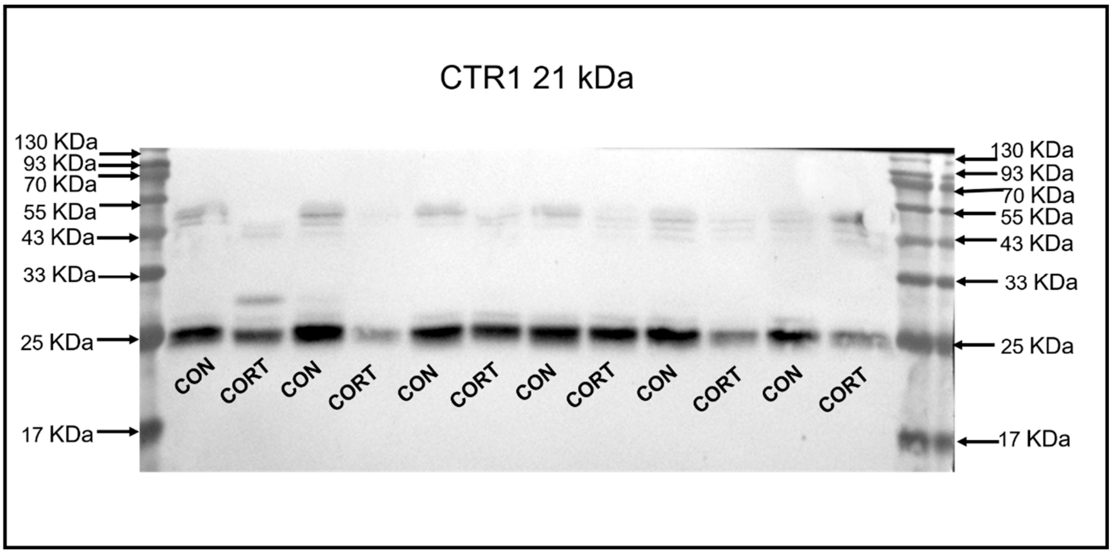
CTR1 21 kDa
130 KDa
93 KDa
70 KDa
55 KDa
43 KDa
33 KDa
25 KDa
17 KDa
CON CORT CON CORT CON CORT CON CORT CON CORT CON CORT
130 KDa
93 KDa
70 KDa
55 KDa
43 KDa
33 KDa
25 KDa
17 KDa

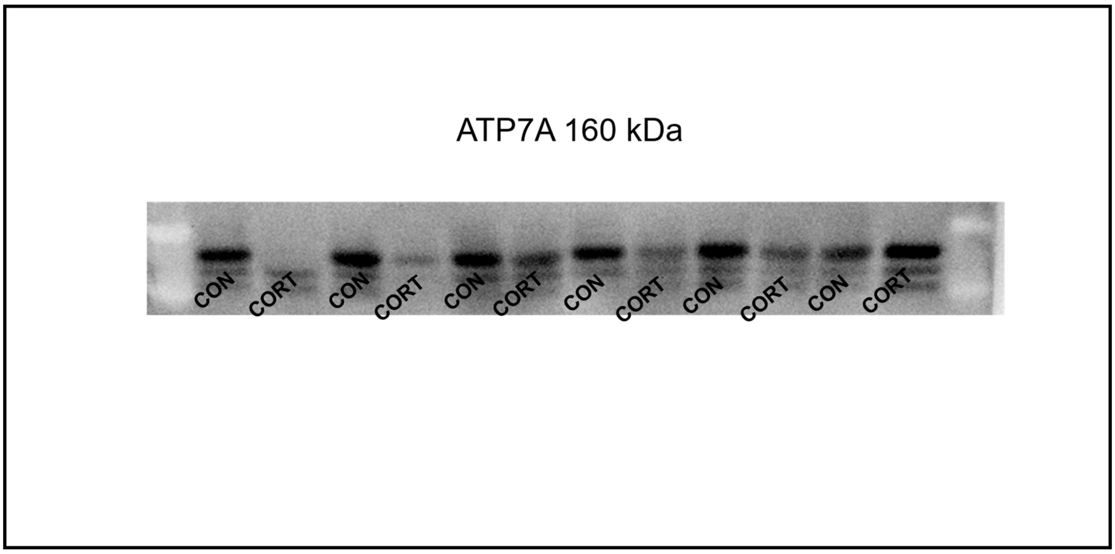
ATP7A 160 kDa
CON CORT CON CORT CON CORT CON CORT CON CORT CON CORT

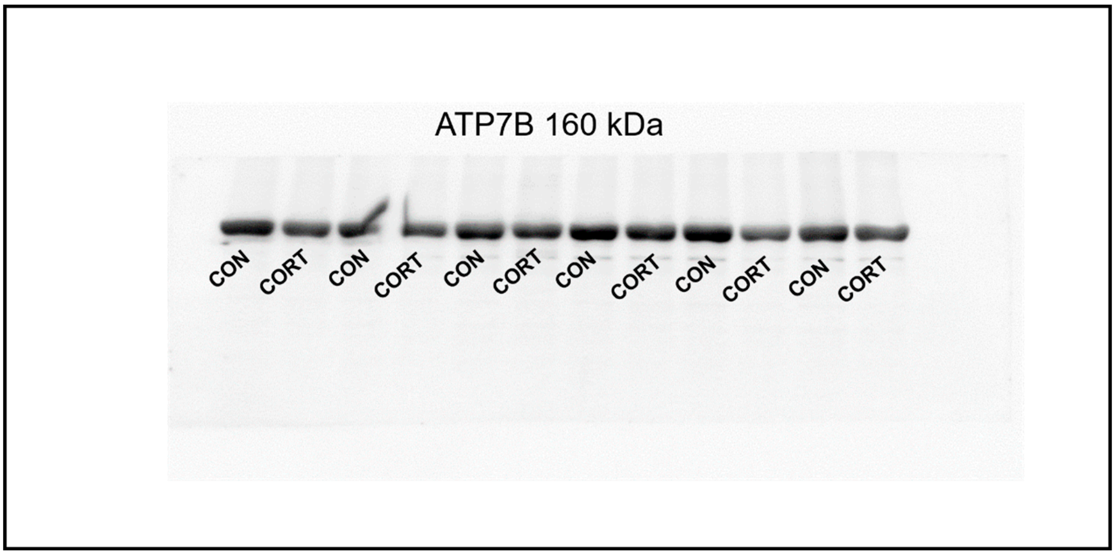
ATP7B 160 kDa
CON CORT CON CORT CON CORT CON CORT CON CORT CON CORT

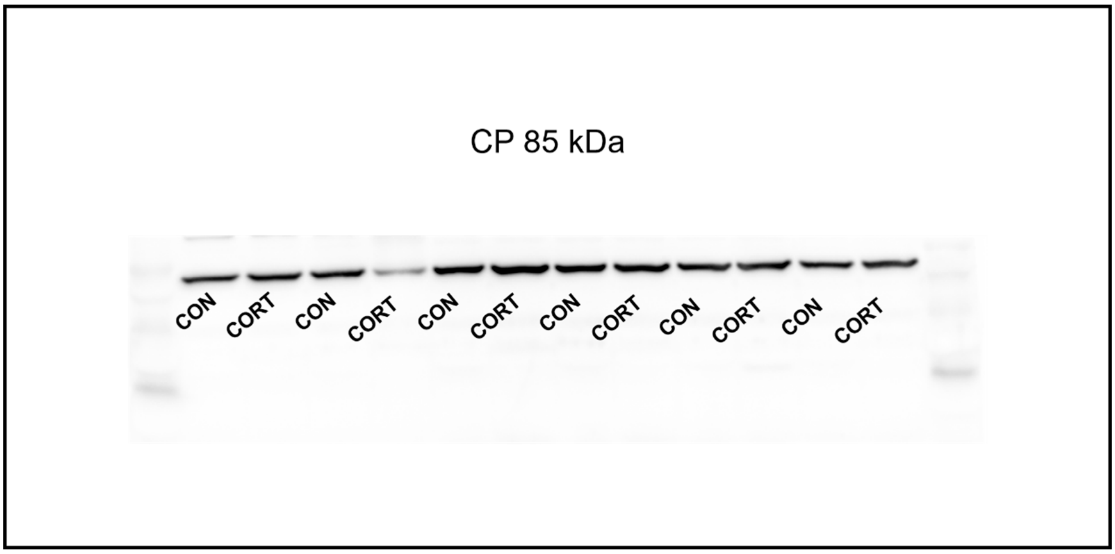
CP 85 kDa
CON CORT CON CORT CON CORT CON CORT CON CORT CON CORT

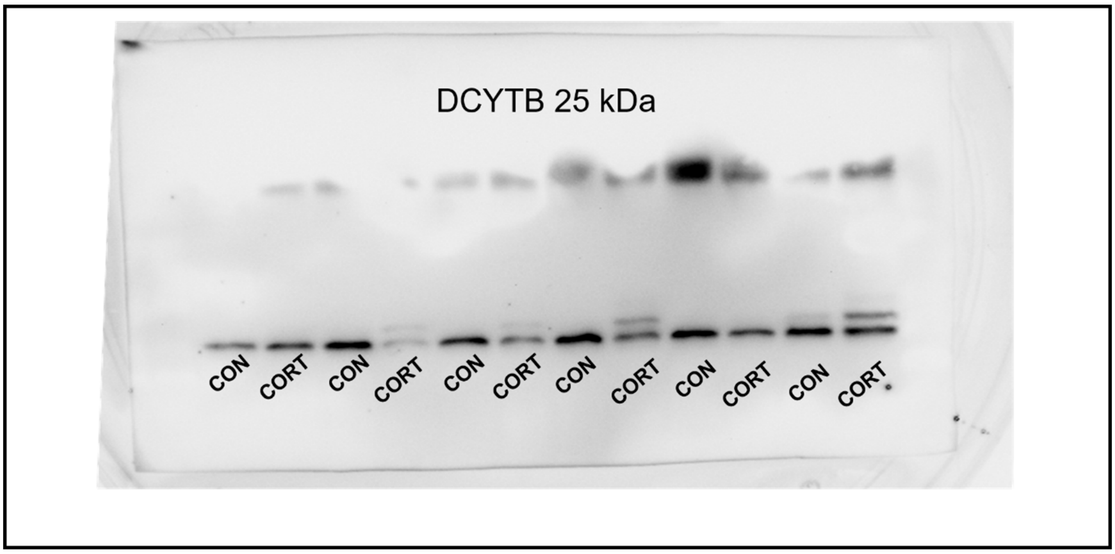
DCYTB 25 kDa
CON CORT CON CORT CON CORT CON CORT CON CORT CON CORT

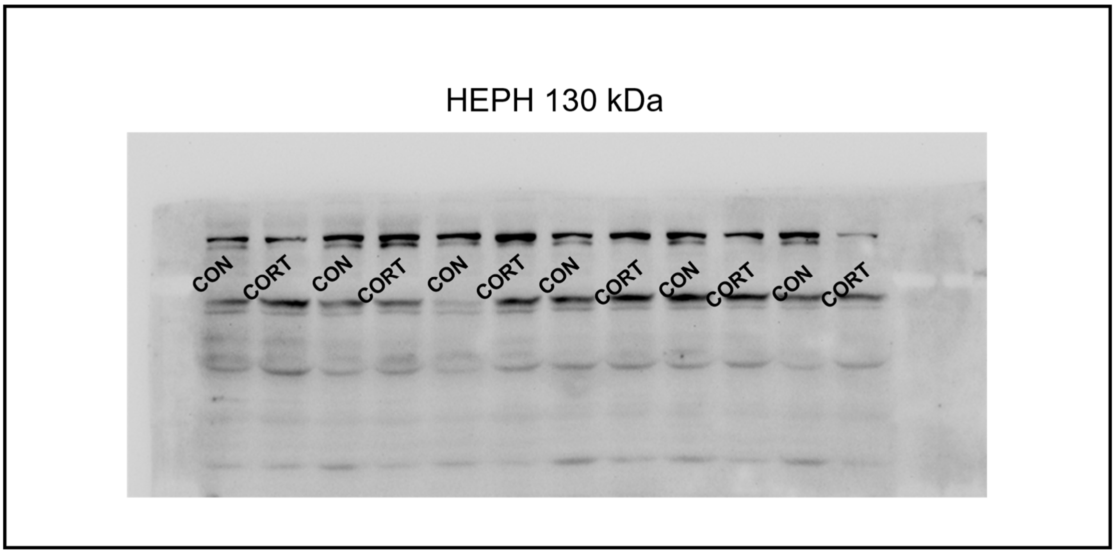
HEPH 130 kDa
CON CORT CON CORT CON CORT CON CORT CON CORT CON CORT

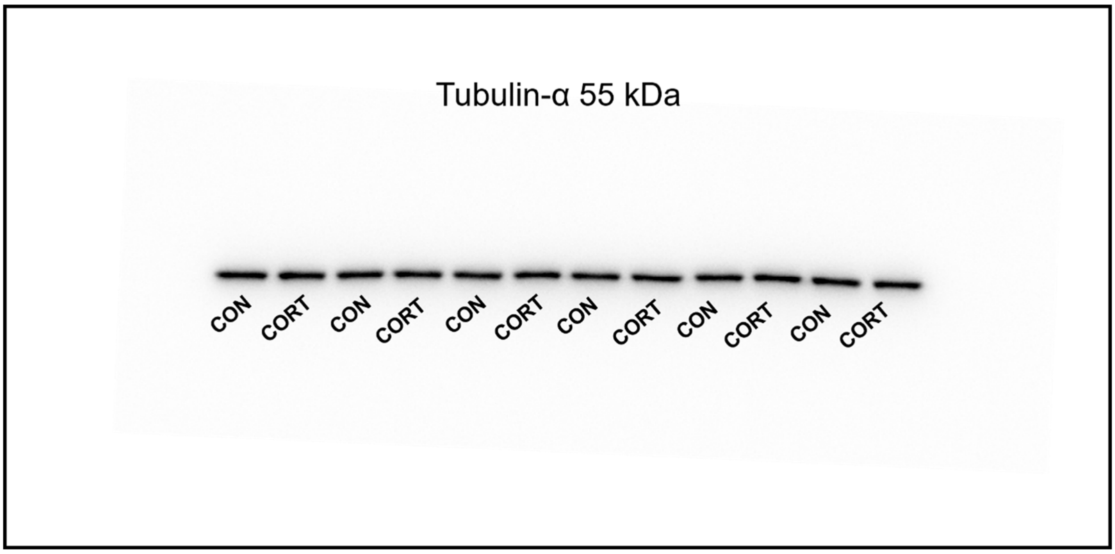

Figure S3A up. Mouse liver H&E staining, the first three pictures are the control, and the last three pictures are the CORT group, n=3

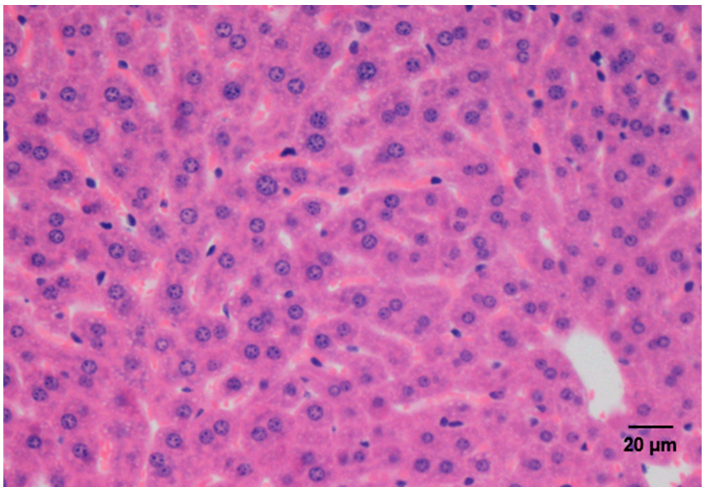

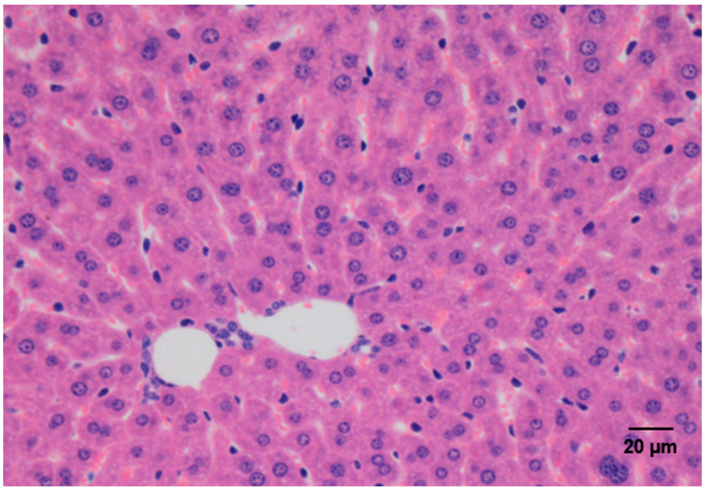

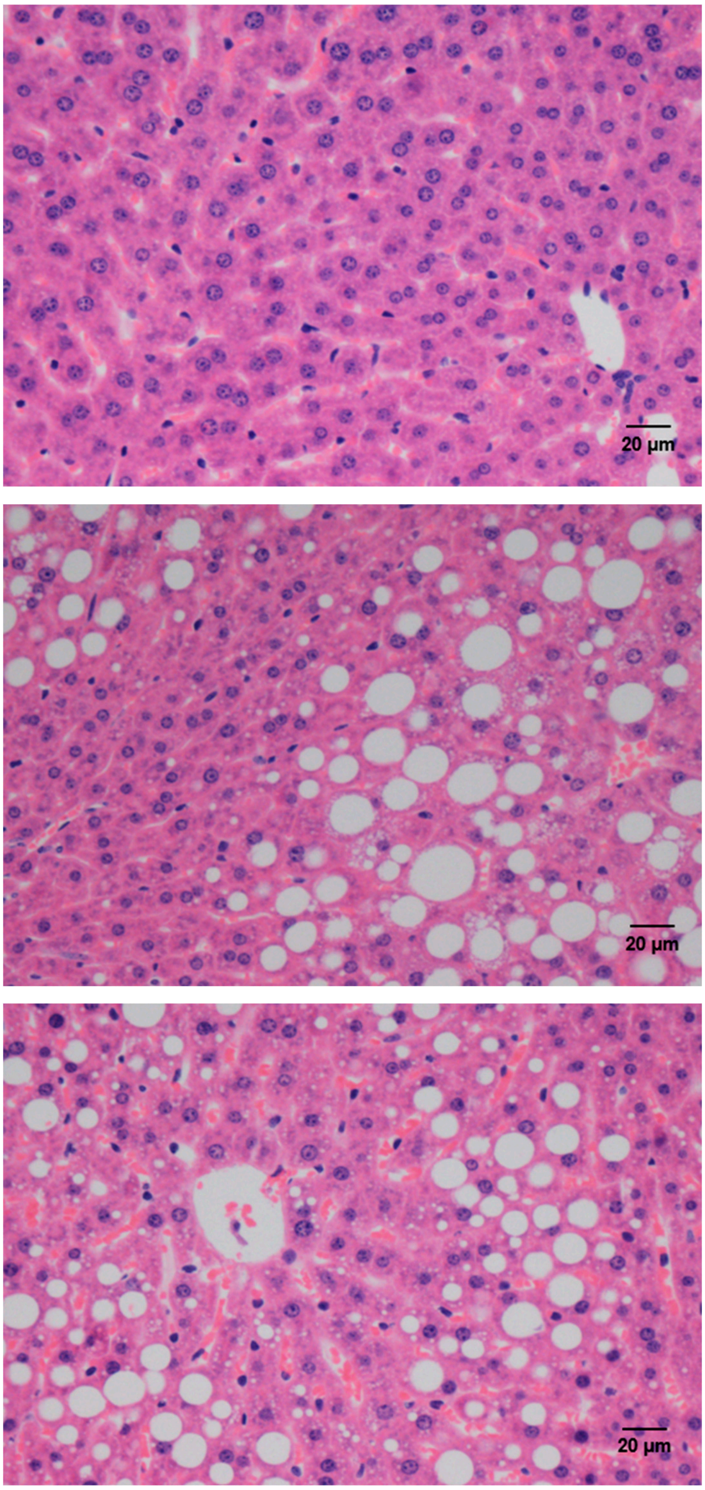
20 μm
20 μm
20 μm

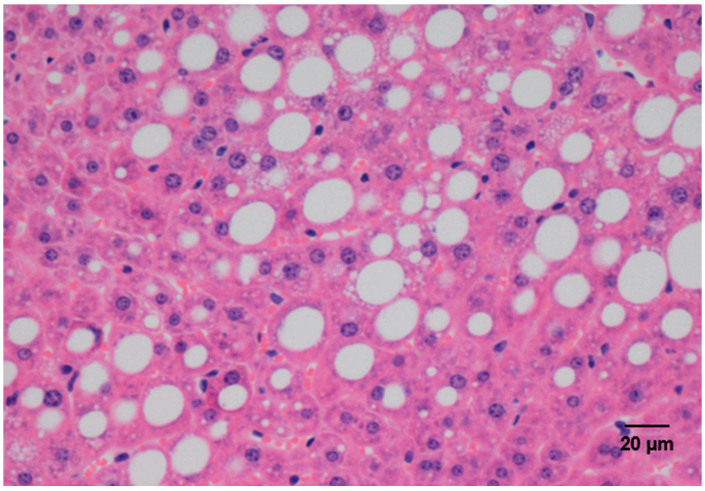

Figure S3A down. Mouse liver metallothionein immunohistochemistry, the first four pictures are the control, and the last four pictures are the CORT group, n=3

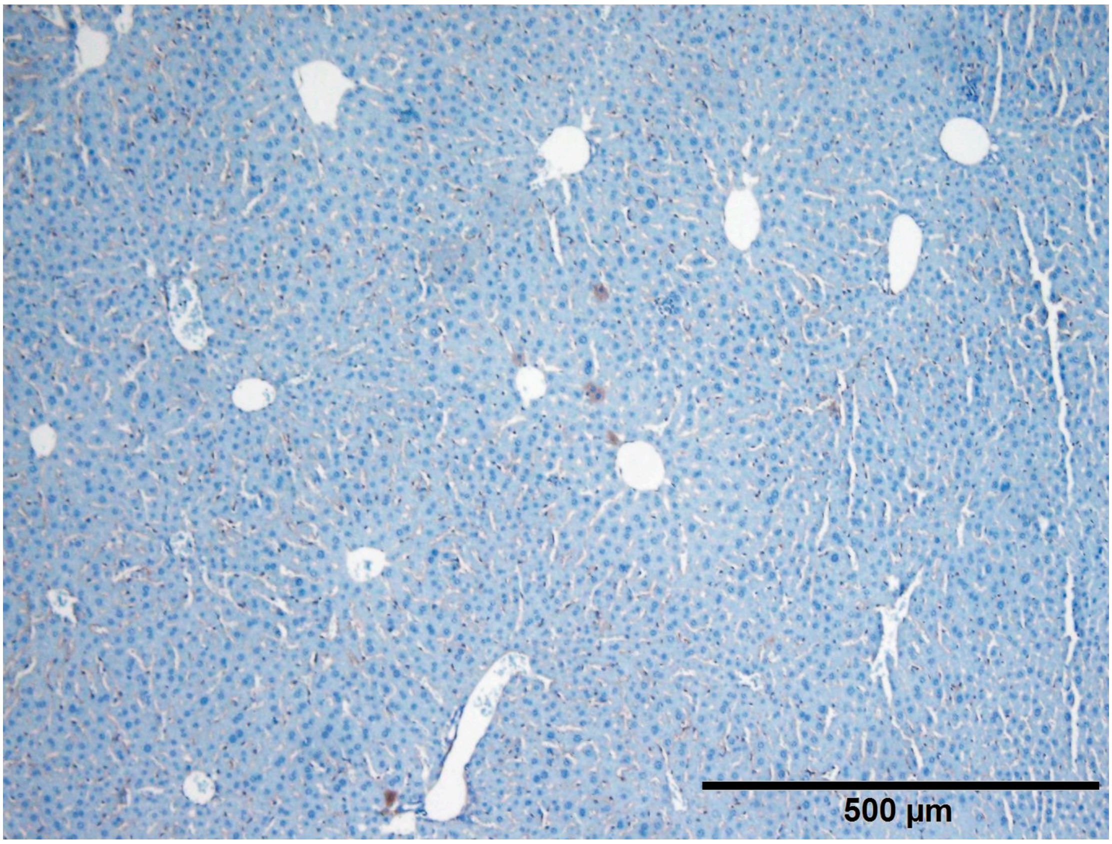

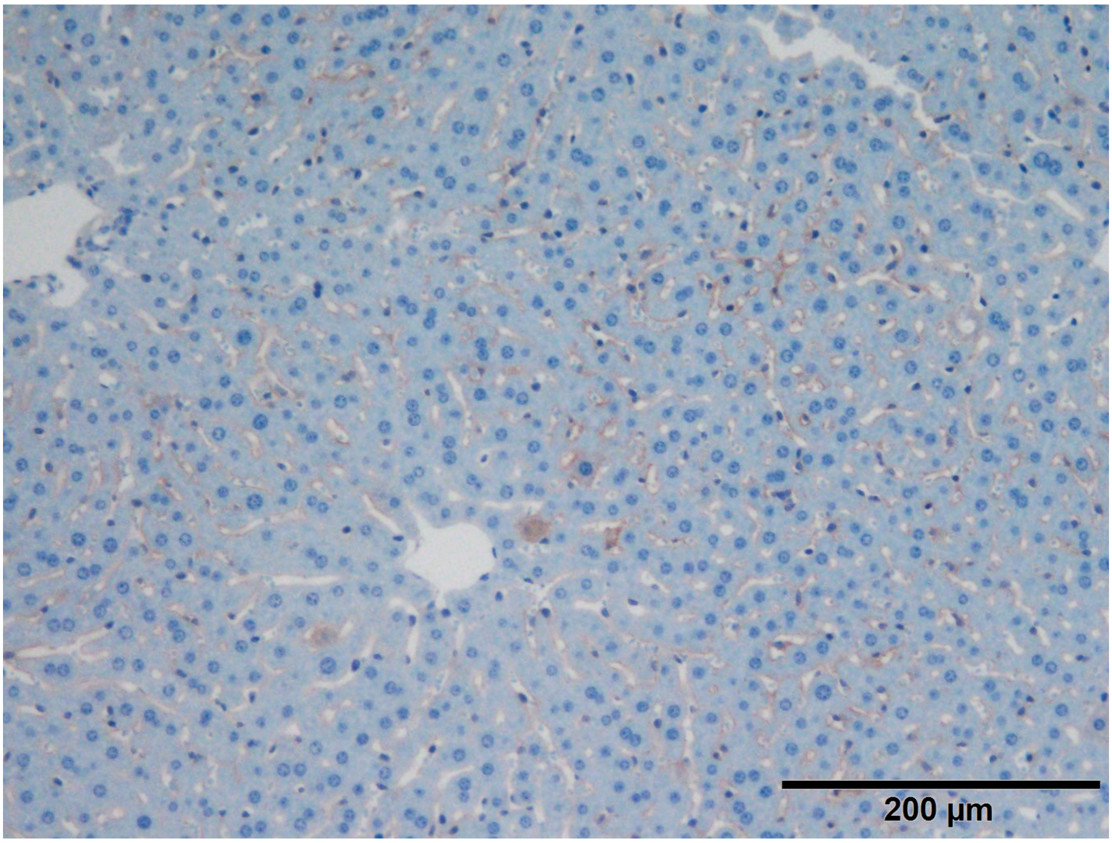
200 µm

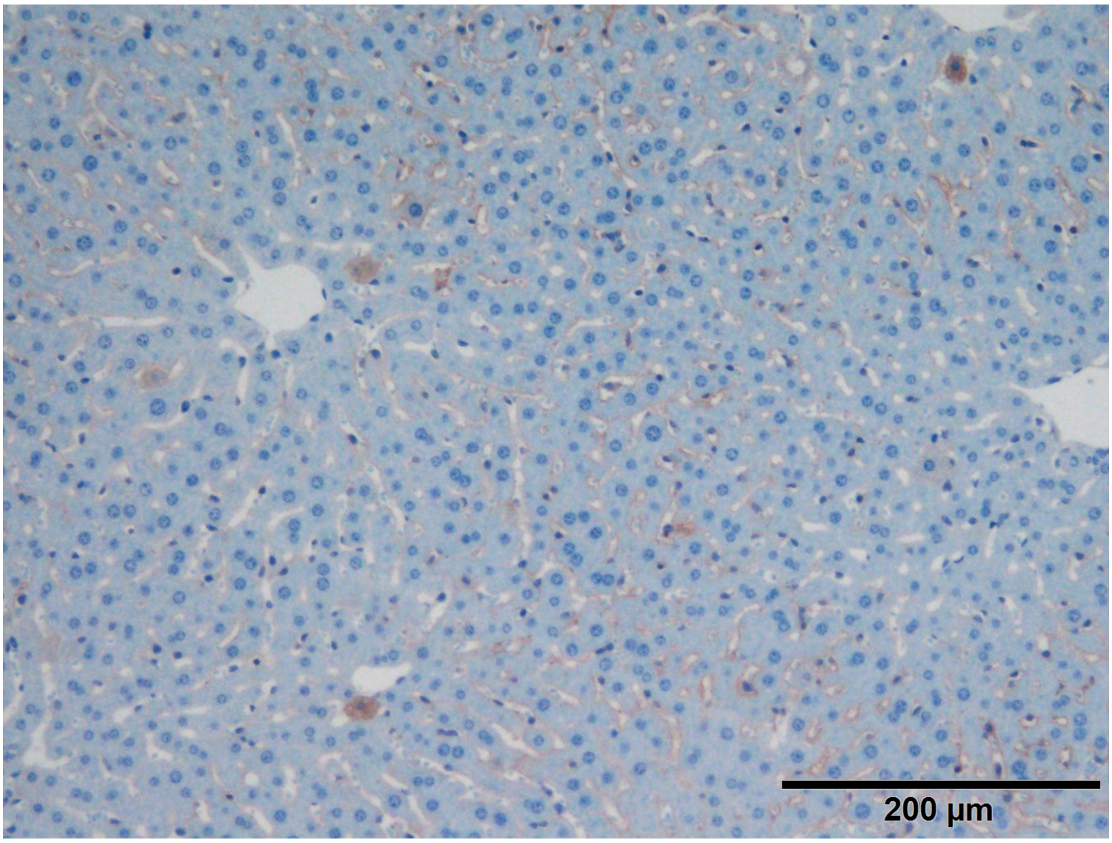
200 µm

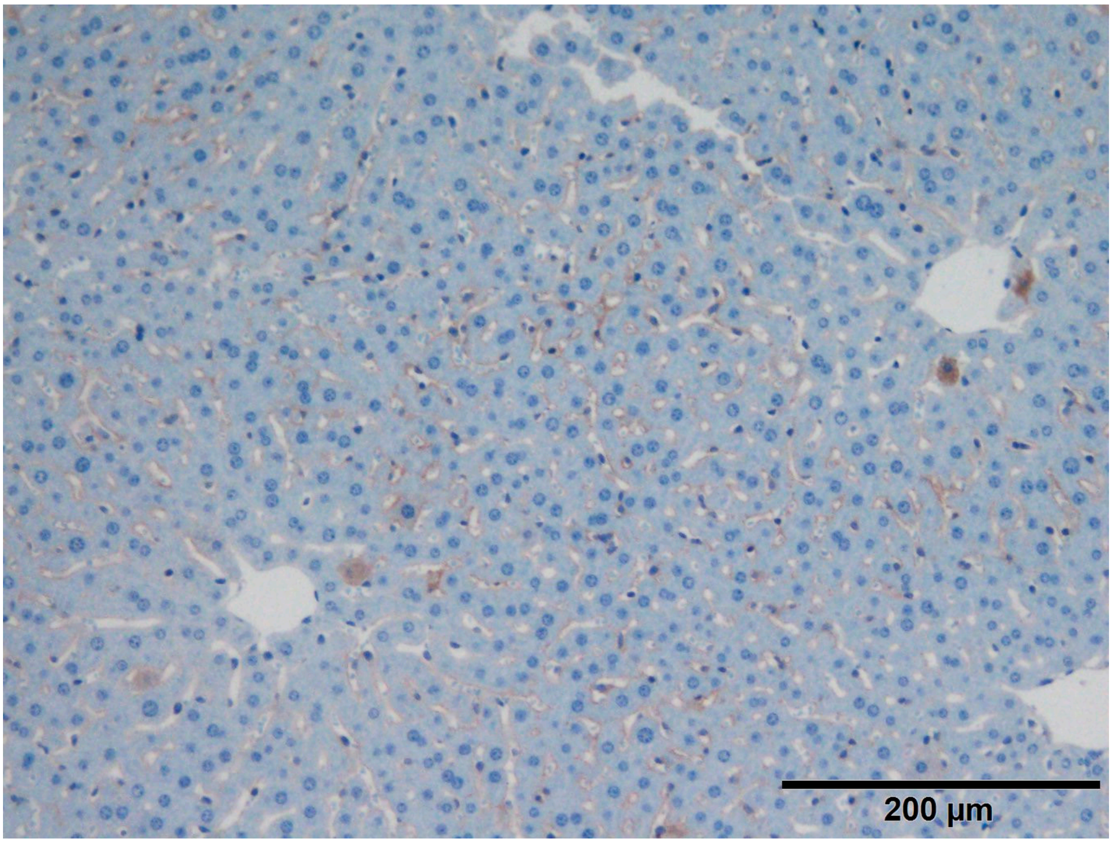
200 µm

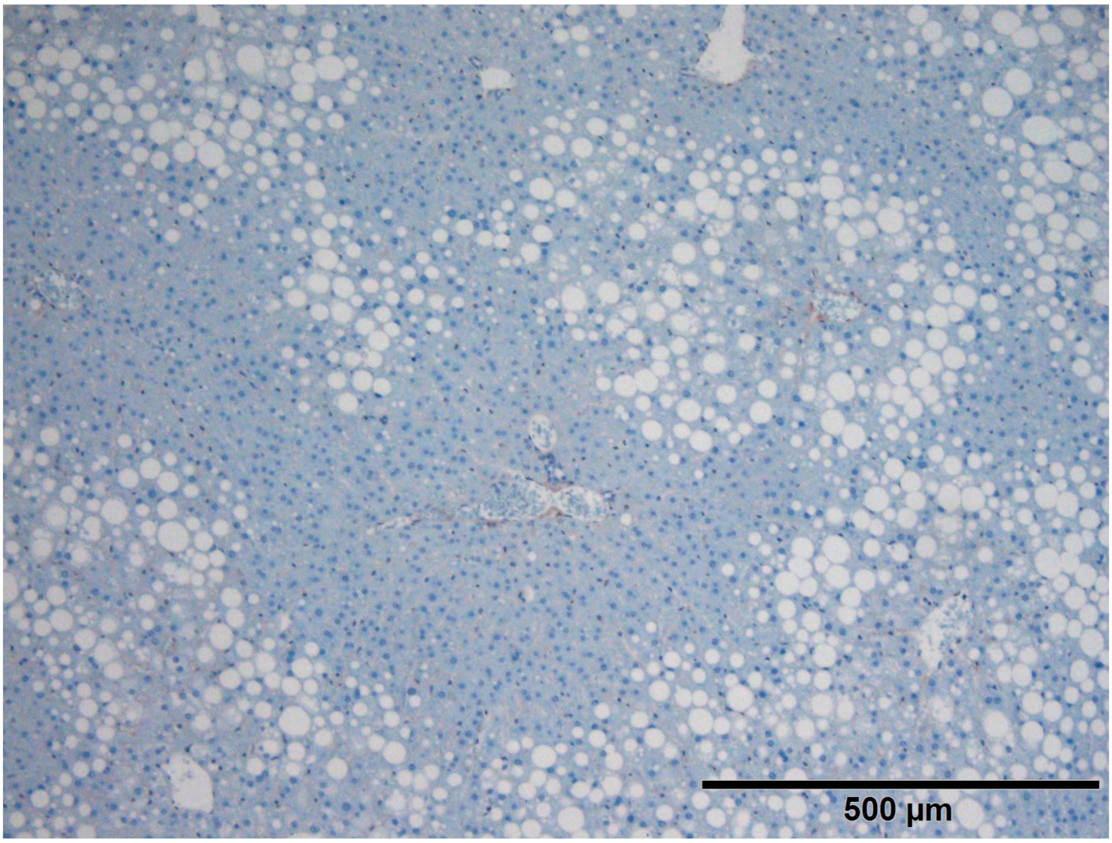
500 µm

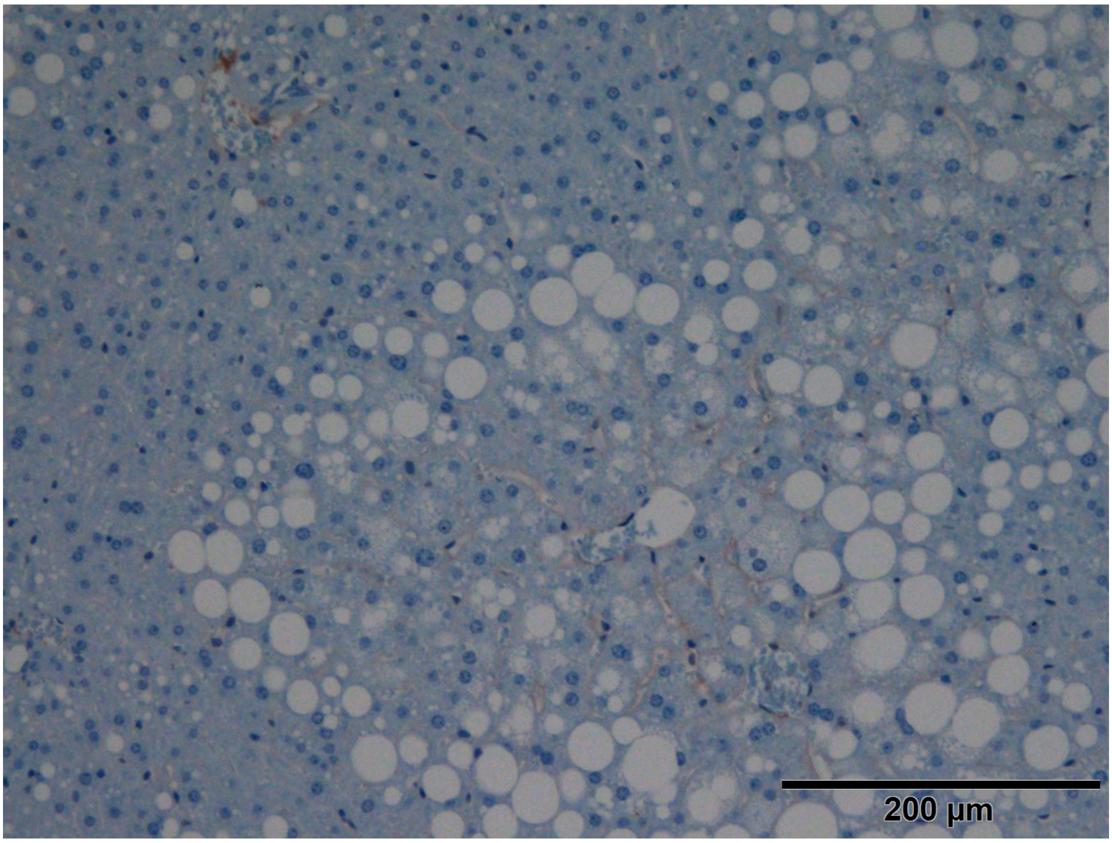
200 µm

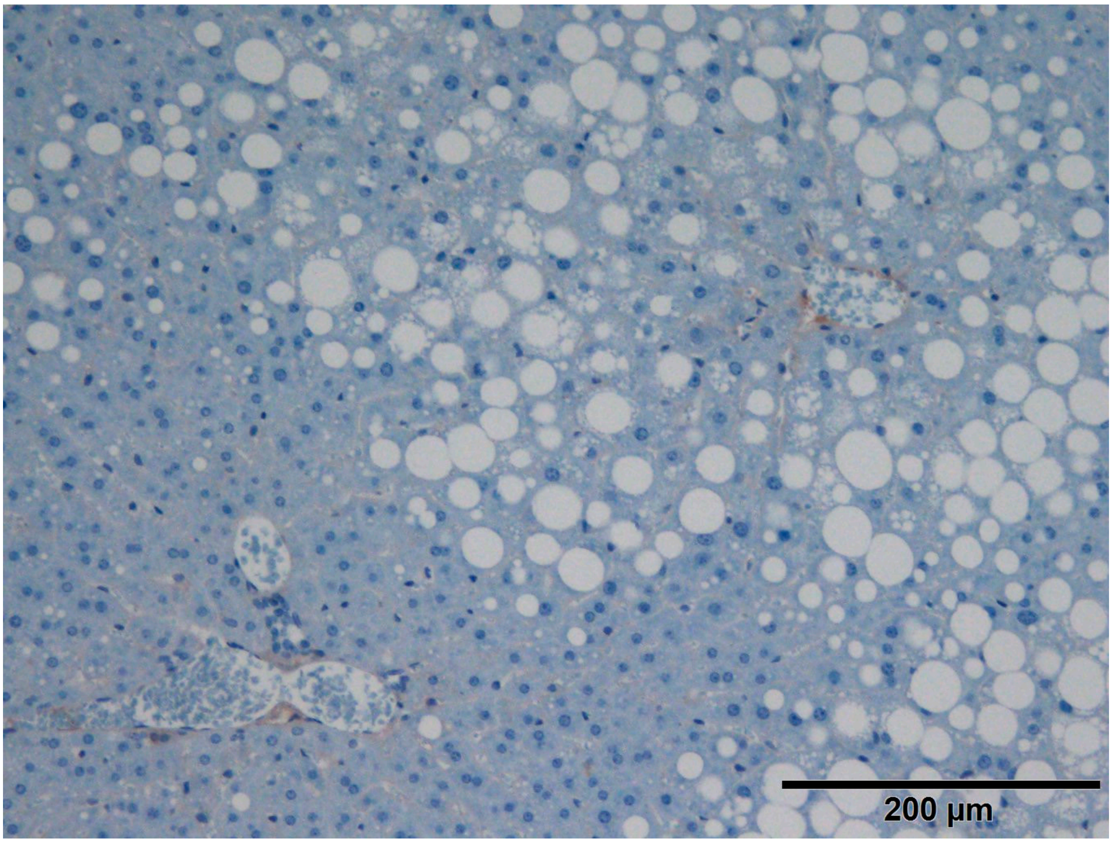
200 µm

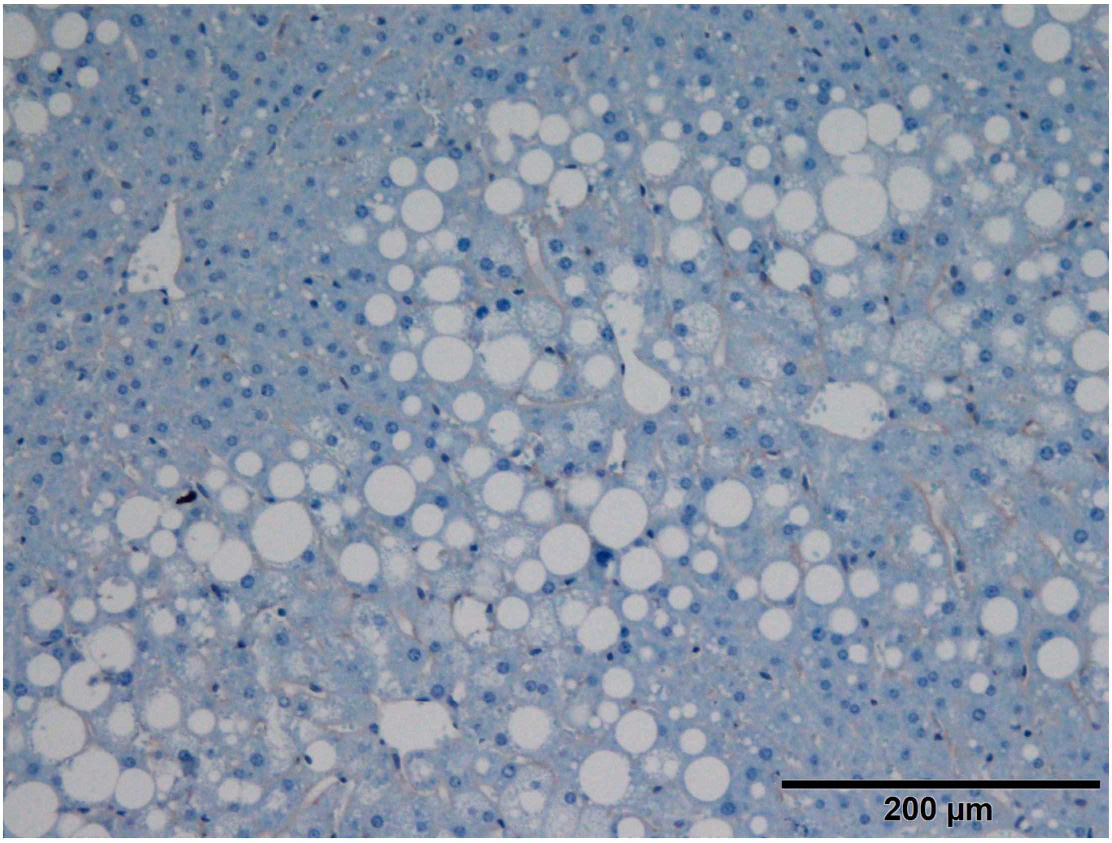

Figure S3D CTR1, ATP7A, ATP7B, DMT1, CP and Tublin-α bands observed in liver of mice.

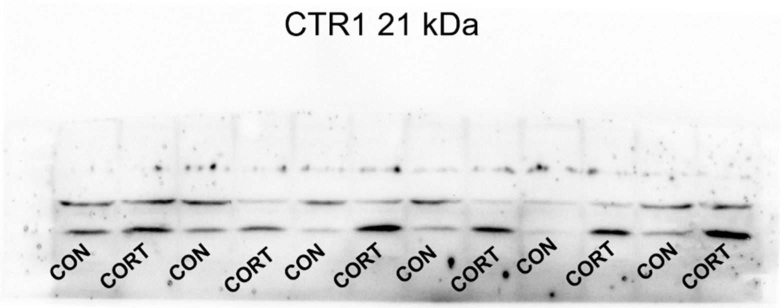

ATP7A 160 kDa

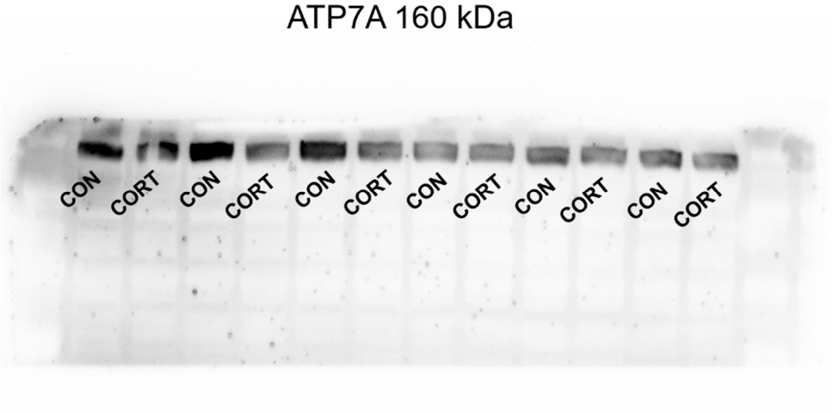

ATP7B 160 kDa

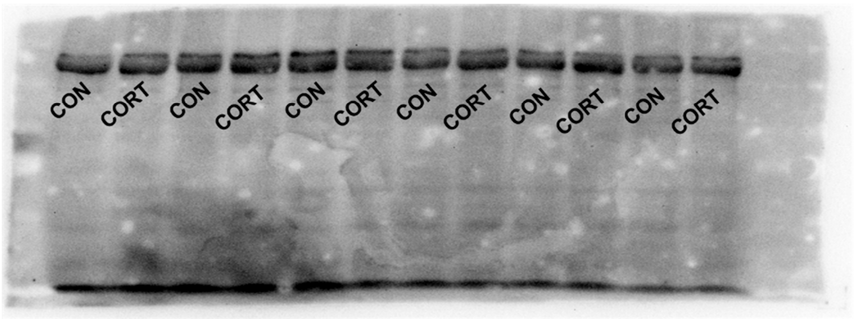

DMT1 62 kDa

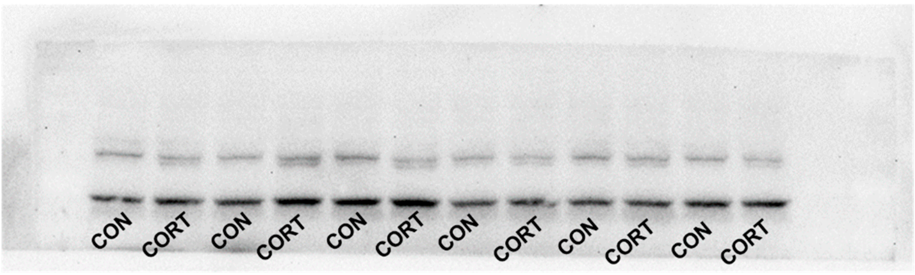

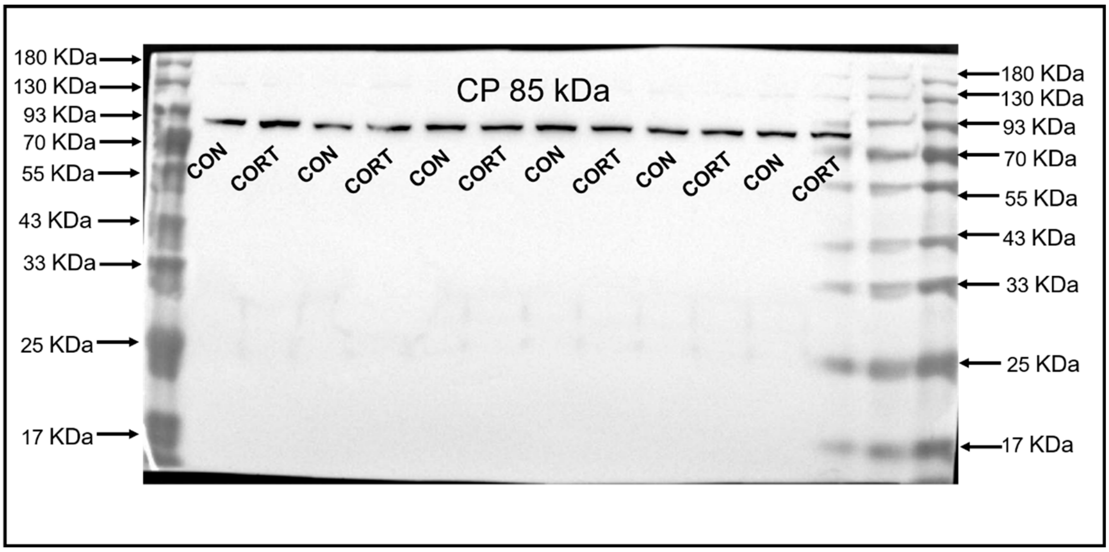

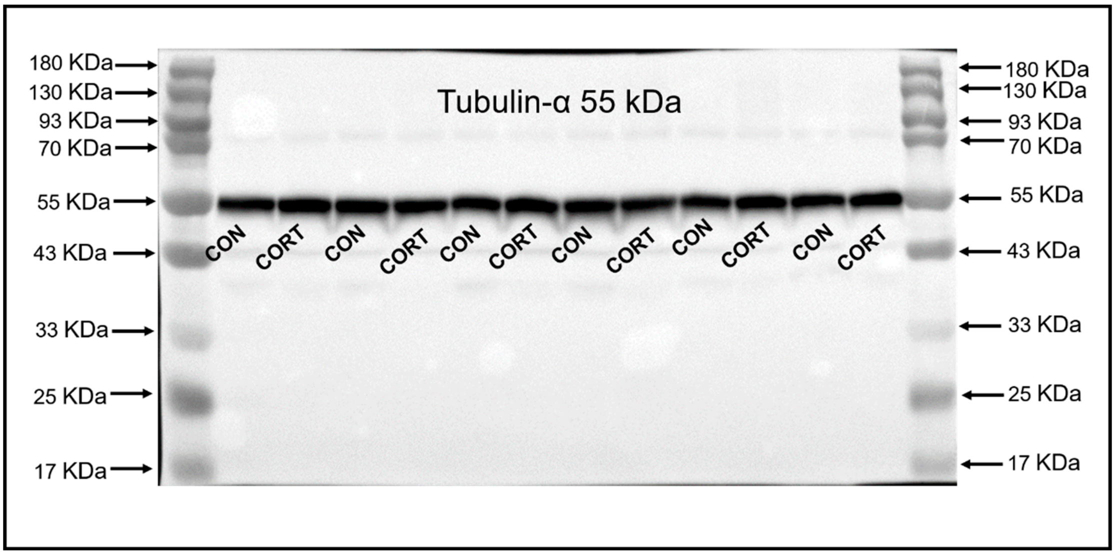

Figure S4A CTR1 and β-Actin bands observed in Caco-2 cells

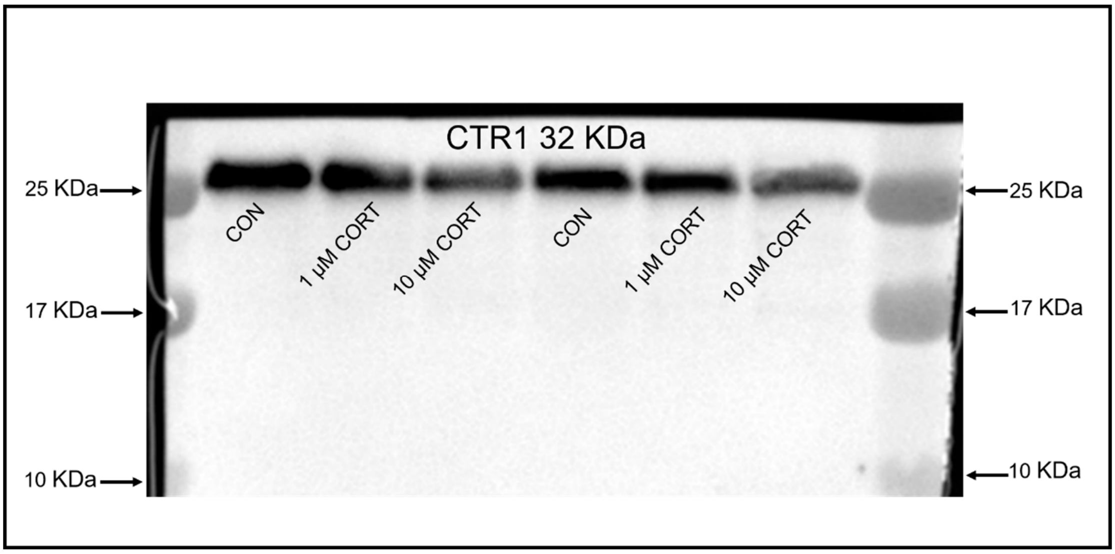

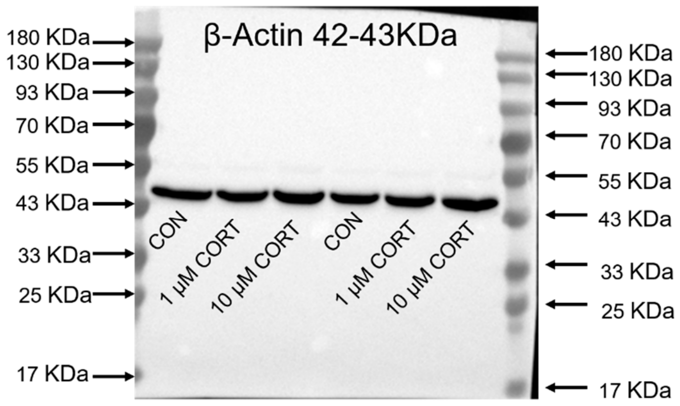

Figure S4B CTR1 and Tubulin-α bands observed in Caco-2 cells

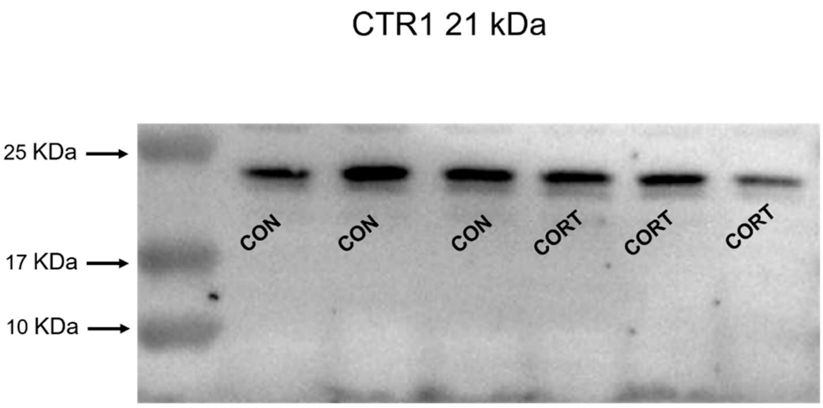

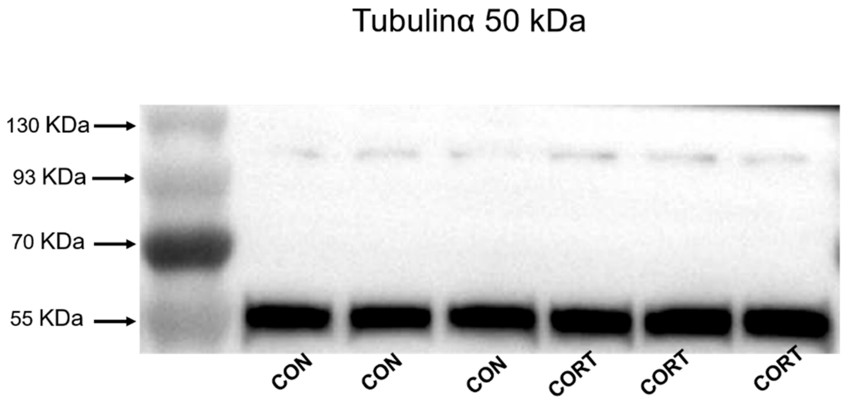

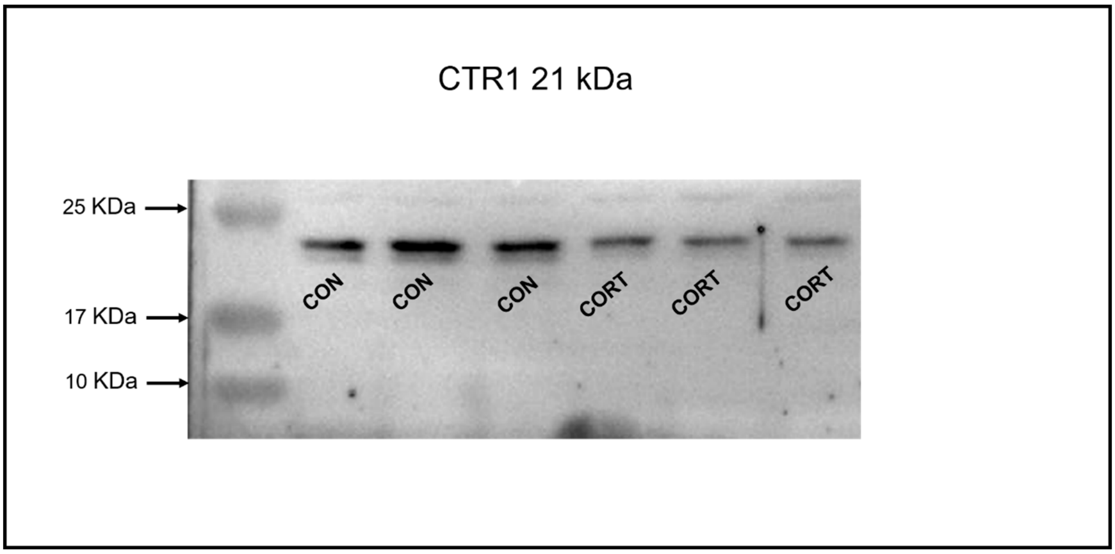

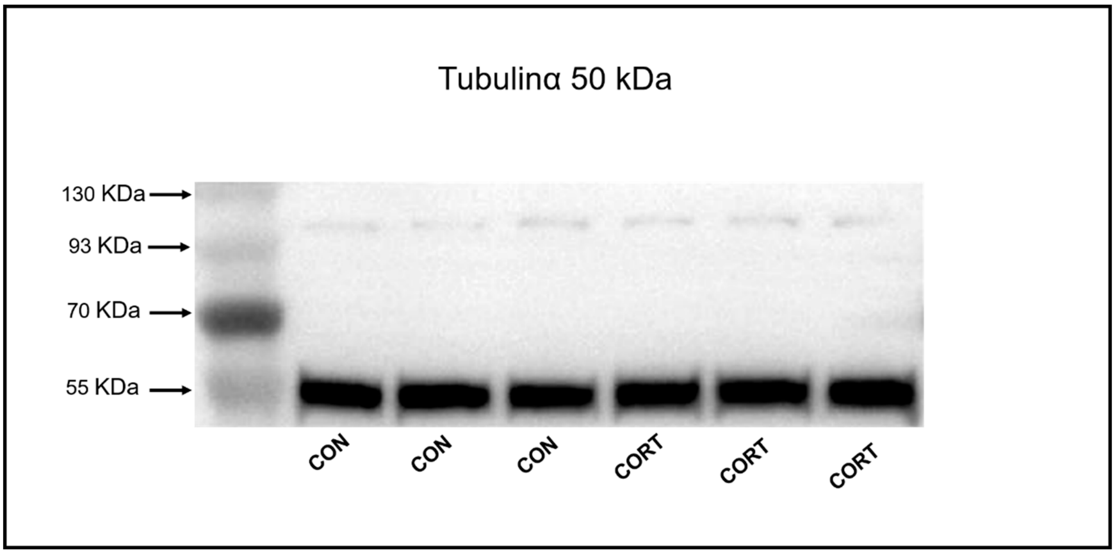

Figure S4C CTR1 and Tubulin-α bands observed in HepG2 cells

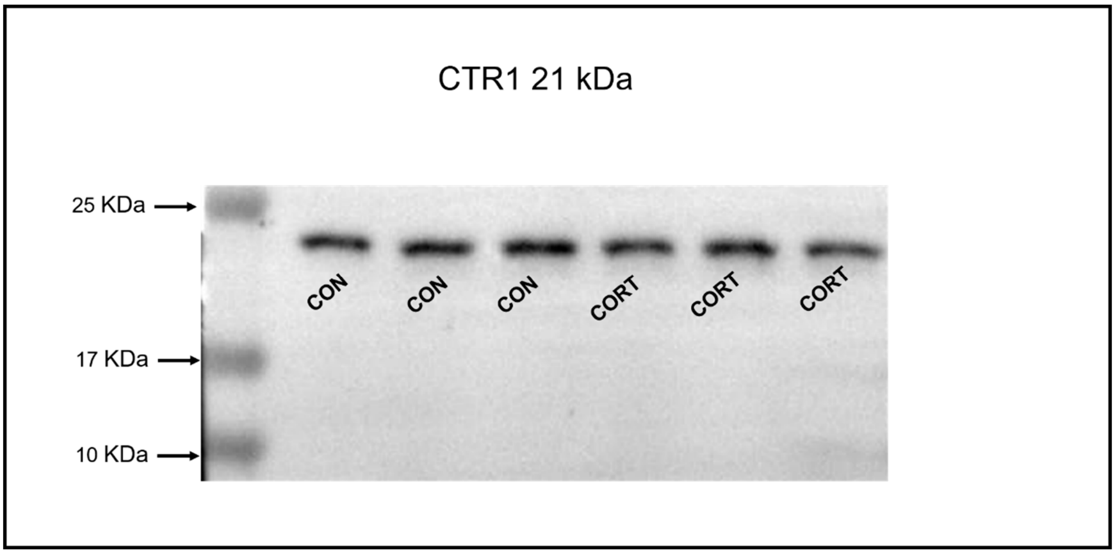

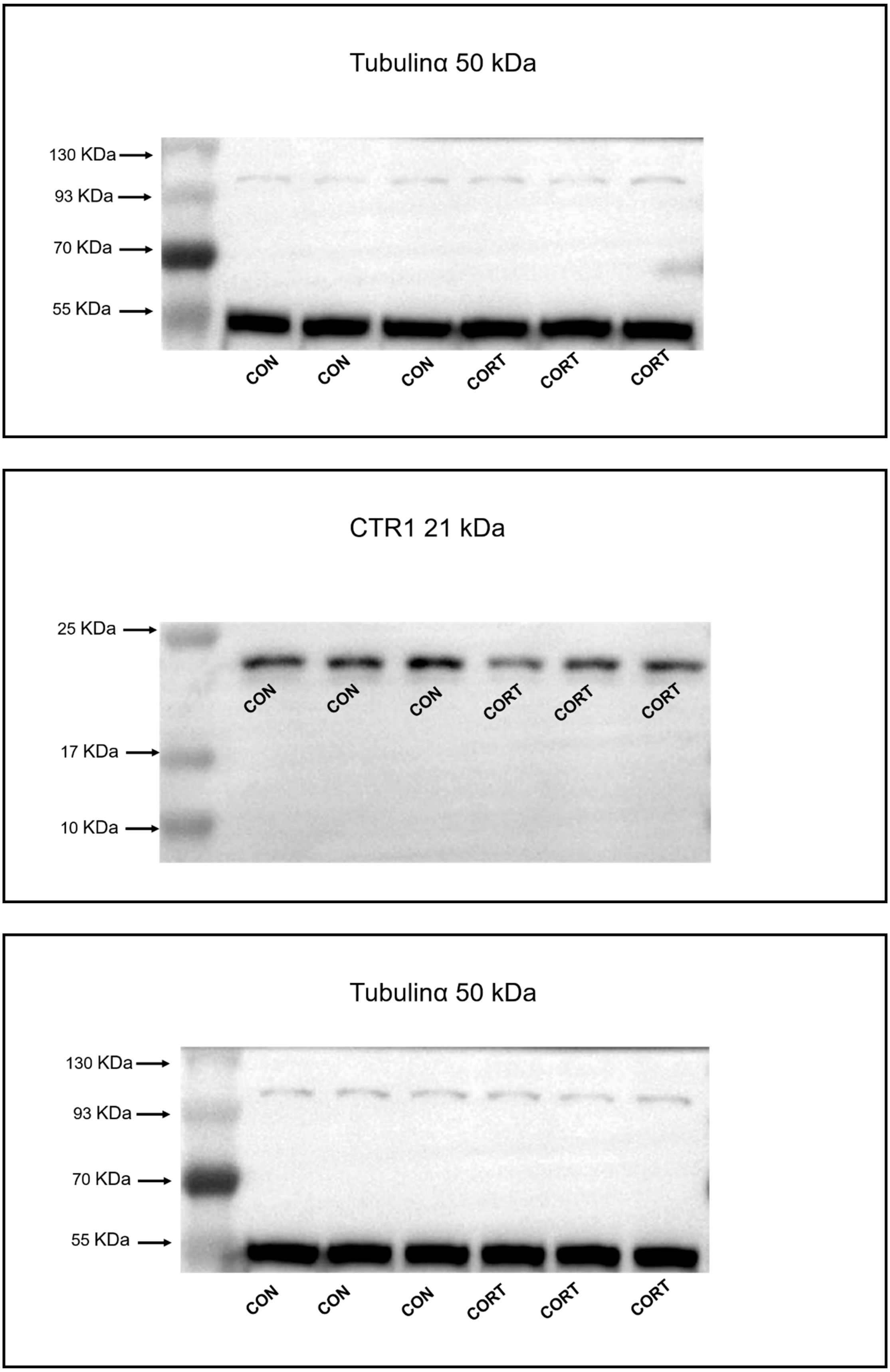

Figure S5A GR, p-GR and β-Actin bands observed in Caco-2 cells

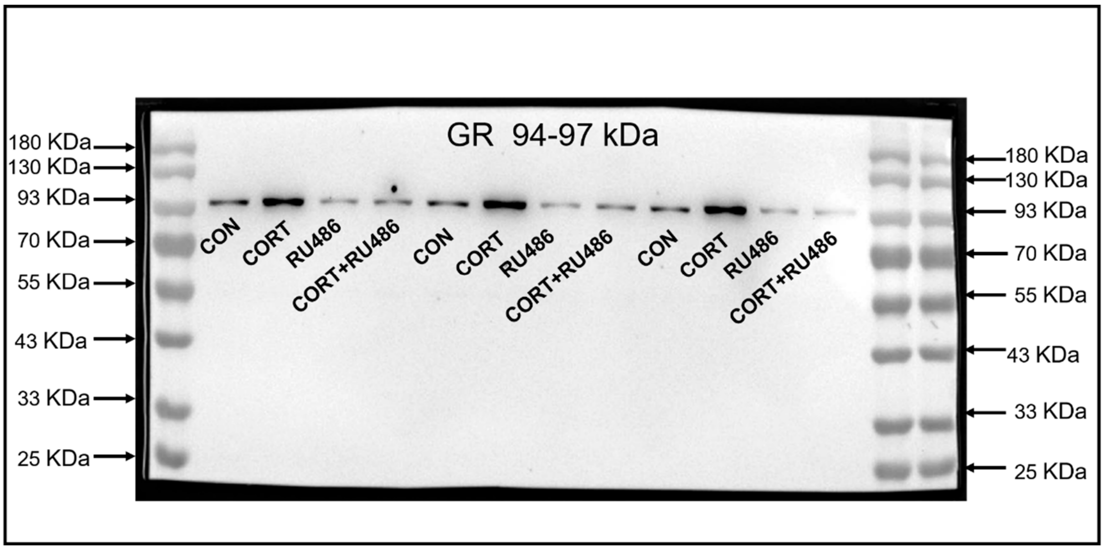

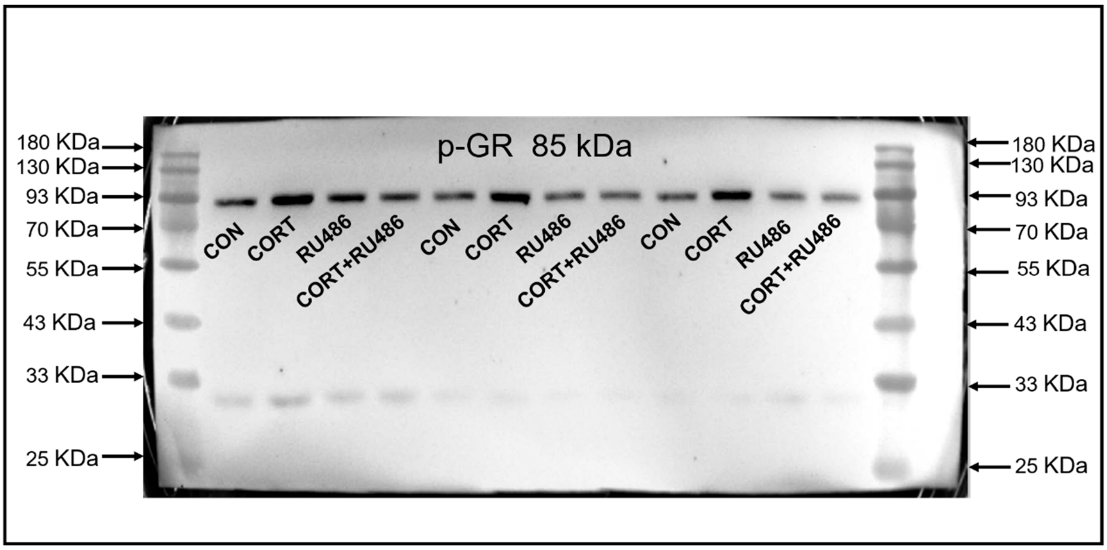

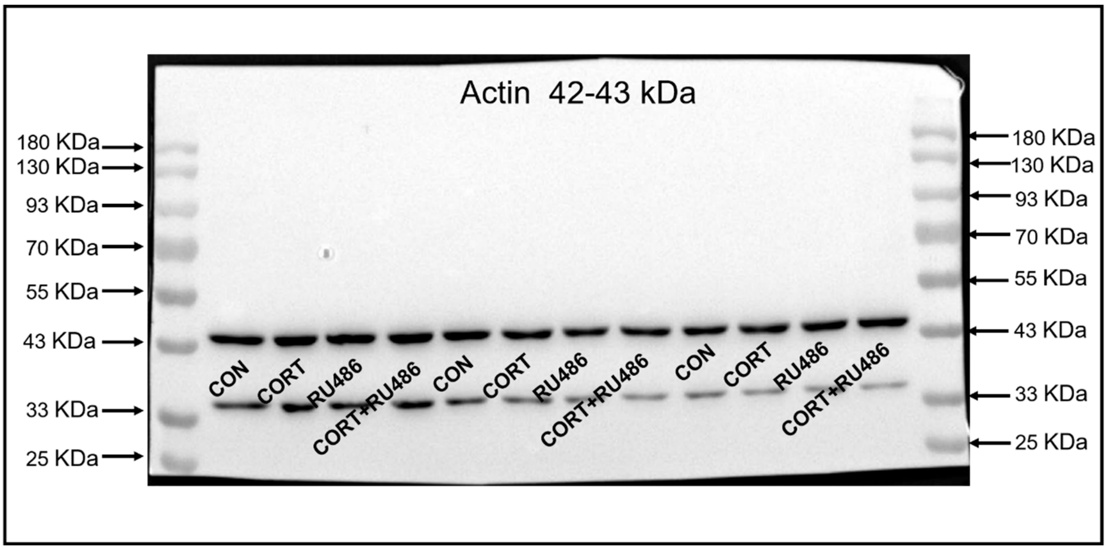

Figure S5B GR, Lamin-β and Tubulin-α bands observed in Caco-2 cells

GR 86 kDa

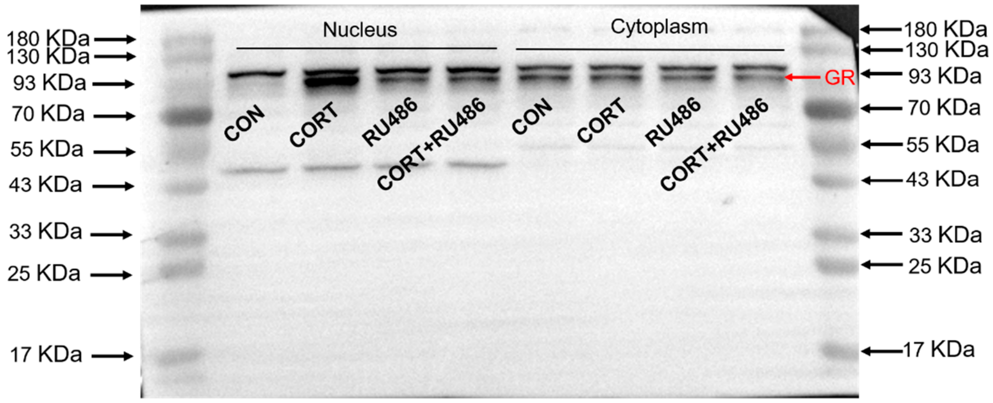

GR 85 kDa

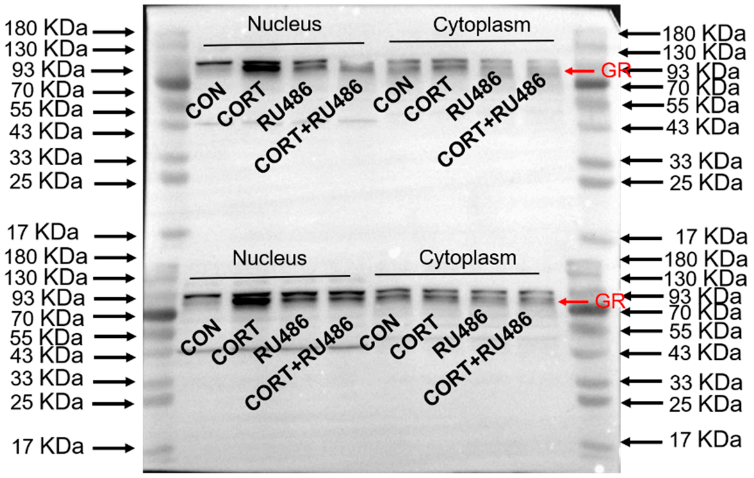

Lamin B1 68 kDa

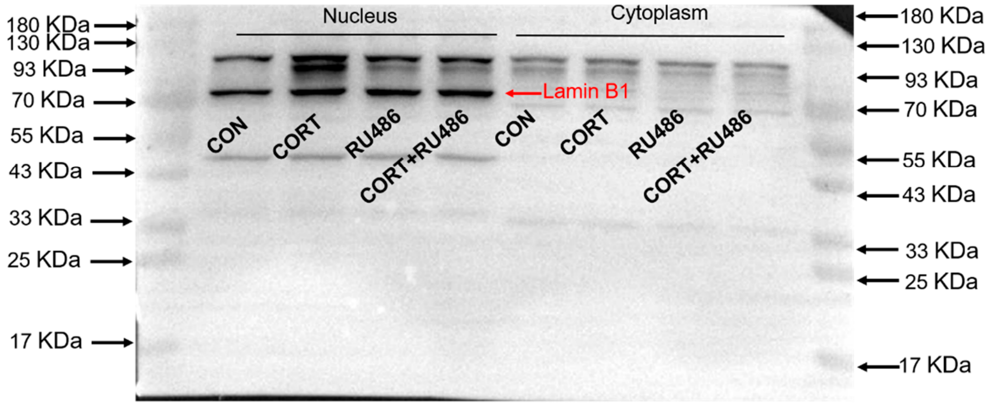

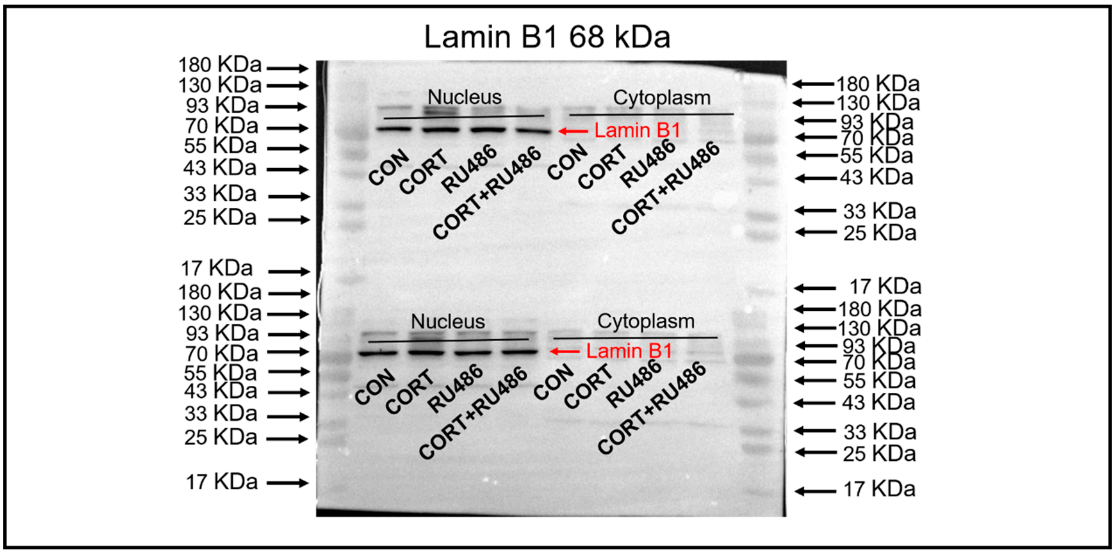

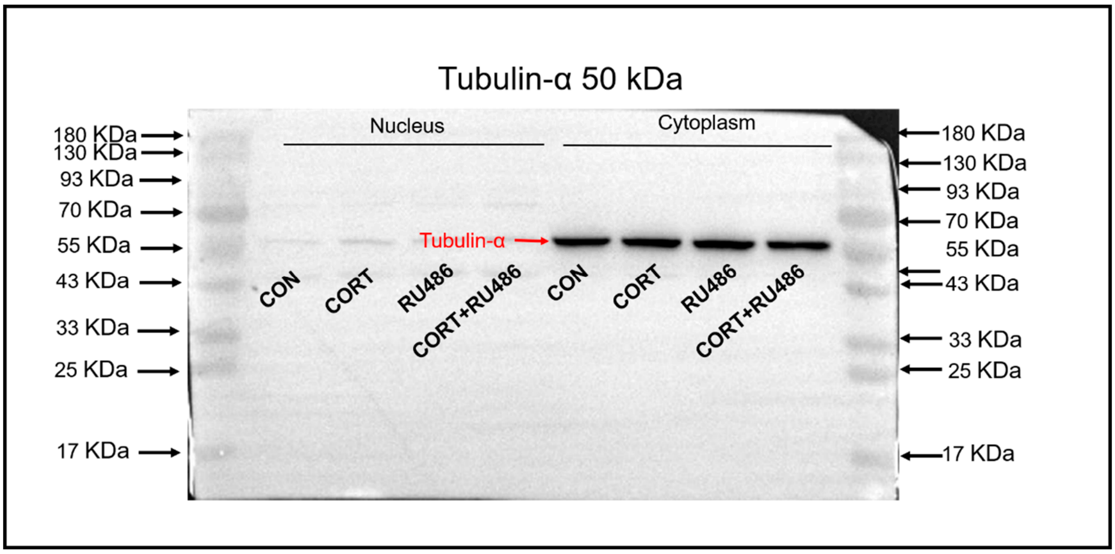

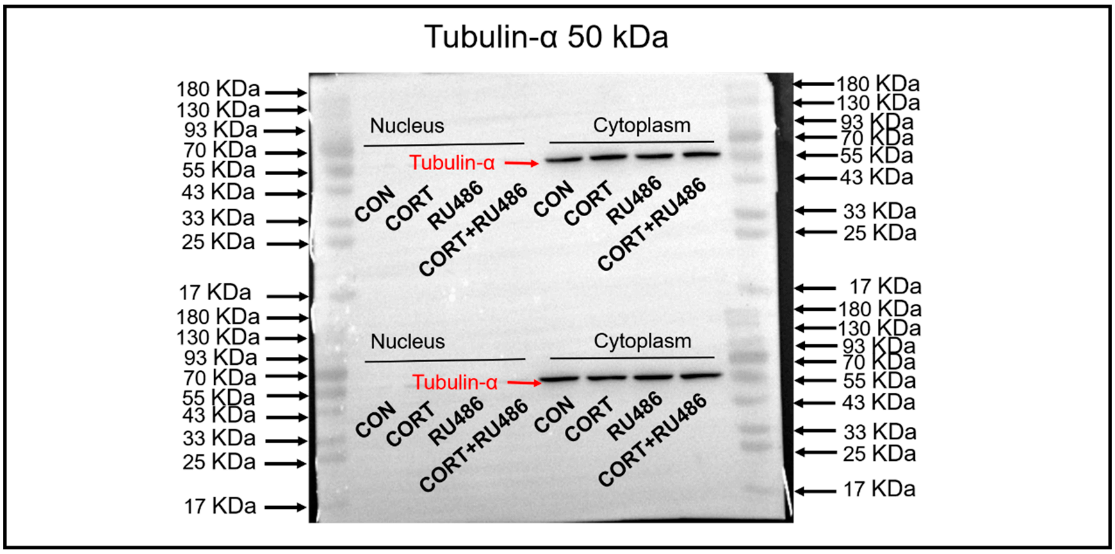

Figure S6B CTR1 and Tubulin-α bands observed in Caco-2 cells

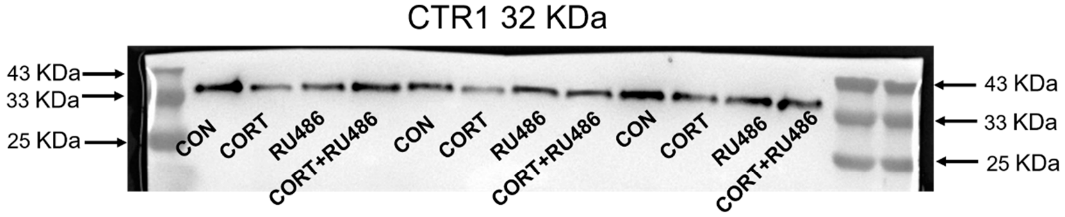
CTR1 32 KDa
43 KDa
33 KDa
25 KDa
CON
CORT
RU486
CORT+RU486
CON
CORT
RU486
CORT+RU486
CON
CORT
RU486
CORT+RU486
43 KDa
33 KDa
25 KDa

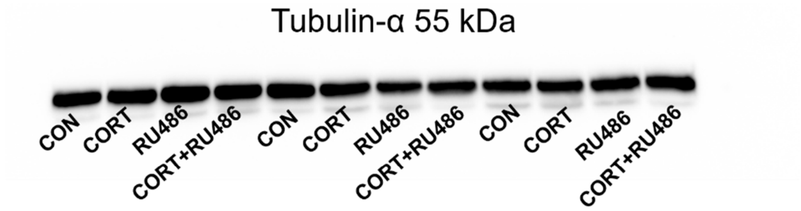
Tubulin-α 55 kDa
CON
CORT
RU486
CORT+RU486
CON
CORT
RU486
CORT+RU486
CON
CORT
RU486
CORT+RU486
